# Nonlinear Transient Permeability in pH-Responsive Bicontinuous Nanospheres

**DOI:** 10.1021/jacs.3c01203

**Published:** 2023-03-30

**Authors:** Wouter P. van den Akker, Hanglong Wu, Pascal L. W. Welzen, Heiner Friedrich, Loai K. E. A. Abdelmohsen, Rolf A. T. M. van Benthem, Ilja K. Voets, Jan C. M. van Hest

**Affiliations:** † Department of Chemistry & Chemical Engineering, Institute for Complex Molecular Systems, Bio-Organic Chemistry, Eindhoven University of Technology, Helix, P.O. Box 513, 5600MB Eindhoven, The Netherlands; ‡ Department of Chemistry & Chemical Engineering, Self-Organizing Soft Matter, Eindhoven University of Technology, P.O. Box 513, 5600MB Eindhoven, The Netherlands; § Department of Chemistry & Chemical Engineering, Laboratory of Physical Chemistry and Center for Multiscale Electron Microscopy, Eindhoven University of Technology, P.O. Box 513, 5600MB Eindhoven, The Netherlands; ∥ Energy Transition Center Amsterdam, Grasweg 31, 1031HW Amsterdam, The Netherlands

## Abstract

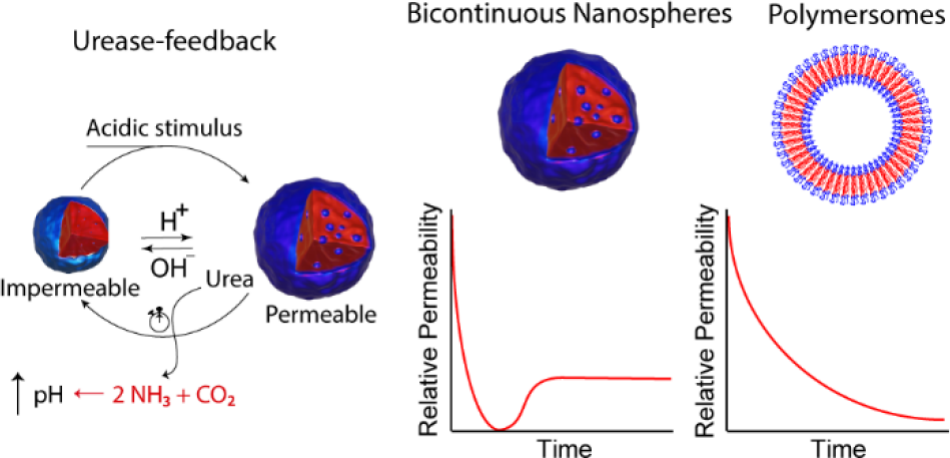

We demonstrate the construction of pH-responsive bicontinuous nanospheres (BCNs) with nonlinear transient permeability and catalytic activity. The BCNs were assembled from amphiphilic block copolymers comprising pH-responsive groups and were loaded with the enzymes urease and horseradish peroxidase (HRP). A transient membrane permeability switch was introduced by employing the well-known pH-increasing effect of urease upon conversion of urea to ammonia. As expected, the coencapsulated HRP displayed a transiently regulated catalytic output profile upon addition of urea, with no significant product formation after the pH increase. This transient process displayed a nonlinear “dampening” behavior, induced by a decrease in membrane permeability as a result of significant local ammonia production. Furthermore, the catalytic output of HRP could be modulated by addition of different amounts of urea or by altering the buffer capacity of the system. Finally, this nonlinear dampening effect was not observed in spherical polymersomes, even though the membrane permeability could also be inhibited by addition of urea. The specific BCN morphology therefore allows to optimally control catalytic processes by pH changes in the nanoreactor microenvironment compared to bulk conditions due to its unique permeability profile.

## Introduction

Stimuli-responsive materials are a well-investigated class of materials which have found widespread applications as for example (soft) actuators, sensors, and drug delivery vehicles.^1,2^ The design window is large, ranging from nano/microgels^3,4^ and polymersomes^5^ to more surface-based systems like polymer brushes^6^ and liquid crystal networks.^7,8^ These materials typically contain functional moieties that are able to respond to environmental cues (e.g., temperature, light, or pH). This responsiveness is often unidirectional, which means that for the materials to change their features back to their starting conditions a reverse stimulus has to be applied. A special class of responsive materials has the ability to revert the response via an internally triggered mechanism, which gives them transient features and makes them adaptive. Transient behavior has been an important topic of investigation in the field of systems chemistry, where a range of concepts, such as the activation or deactivation of molecular buildings blocks, have been developed to transiently control the state of a system, for example its assembly into fibers, vesicles, hydrogels, or higher order colloidal assemblies.^9−13^ More recently, these concepts have also been applied to create materials with integrated transient structural and functional features. Transient assembly of nanoreactors showcases the combination of these features; for example, Maiti et al.^14^ demonstrated the assembly of vesicular nanoreactors under the influence of ATP, which, upon hydrolysis over time, caused the vesicles to disassemble.^14^ Additionally, the yield of a nucleophilic aromatic substitution reaction increased significantly while the vesicles were in their assembled state, introducing catalytic functionality. Apart from the transient assembly of nanoreactors, other approaches have been developed in which structural and functional features are combined, such as the work of Sharma et al.^15^, where the addition of sucrose coassembled individual microgels into larger assemblies. The disassembly process was based on an enzymatic cascade producing gluconic acid. In the assembled state acid generation was increased, as the different elements of the cascade were brought in close spatial proximity, making this reaction proceed more effectively. This proximity effect accelerated the disassembly into individual microgels, illustrating a chemo–structural feedback mechanism.^15^

Many self-regulating systems utilize the enzyme urease for their functionality.^16,17^ This enzyme catalyzes the conversion of urea into ammonia, leading to a pH increase. The bell-shaped curve of the activity profile of this enzyme furthermore contributes to its regulatory application potential.^18^ Our group and others have recently developed polymersome nanoreactors, of which the membrane permeability was regulated by a transient pH change.^19−22^ The enzyme horseradish peroxidase (HRP) was encapsulated together with urease in a polymersome of which the bilayer was cross-linked and functionalized by pH-responsive tertiary amine moieties. Upon addition of an acidic solution containing the HRP substrate (ABTS) and urea, the membrane became swollen and both enzymes had access to their substrates, which allowed the reaction to take place until the pH was reverted to conditions at which the tertiary ammonium groups were deprotonated. We define the pH at which the membrane becomes inaccessible for substrate to be pH*. In this case the transient period seemed to be governed by one process, in which a gradual pH increase is connected to a diminished membrane permeability. This, however, is not that obvious. Even at low bulk pH urease can already become active and should lead to a strong local increase in ammonia concentration, and hence pH, at the site of production. As a result, the responsive polymer system should switch its protonation state and permeability early on in the process, which would have a direct consequence on the accessibility of the substrate. We hypothesized that these different stages in transient behavior were not observed for polymersomes, as in this case the hydrophobic barrier was only a reasonably thin bilayer of about 20 nm in thickness. We therefore were interested in investigating if we could further elaborate on the transient effects by employing nanoparticles with increased membrane thickness, for example by employing morphologies that comprise a more extended hydrophobic domain.

Typical structures assembled from amphiphilic block copolymers include spherical micelles, vesicles, and worm-like polymersomes.^23,24^ However, these are all structures with still rather limited overall membrane thickness. In the past, other morphologies have been observed such as bicontinuous nanospheres^25−27^ (BCNs), which have multiple internal, aqueous pore-like structures embedded in a hydrophobic matrix material, creating an overall cumulative barrier, and which would therefore be suitable to study the effects of an extended hydrophobic domain.

**Scheme 1:**
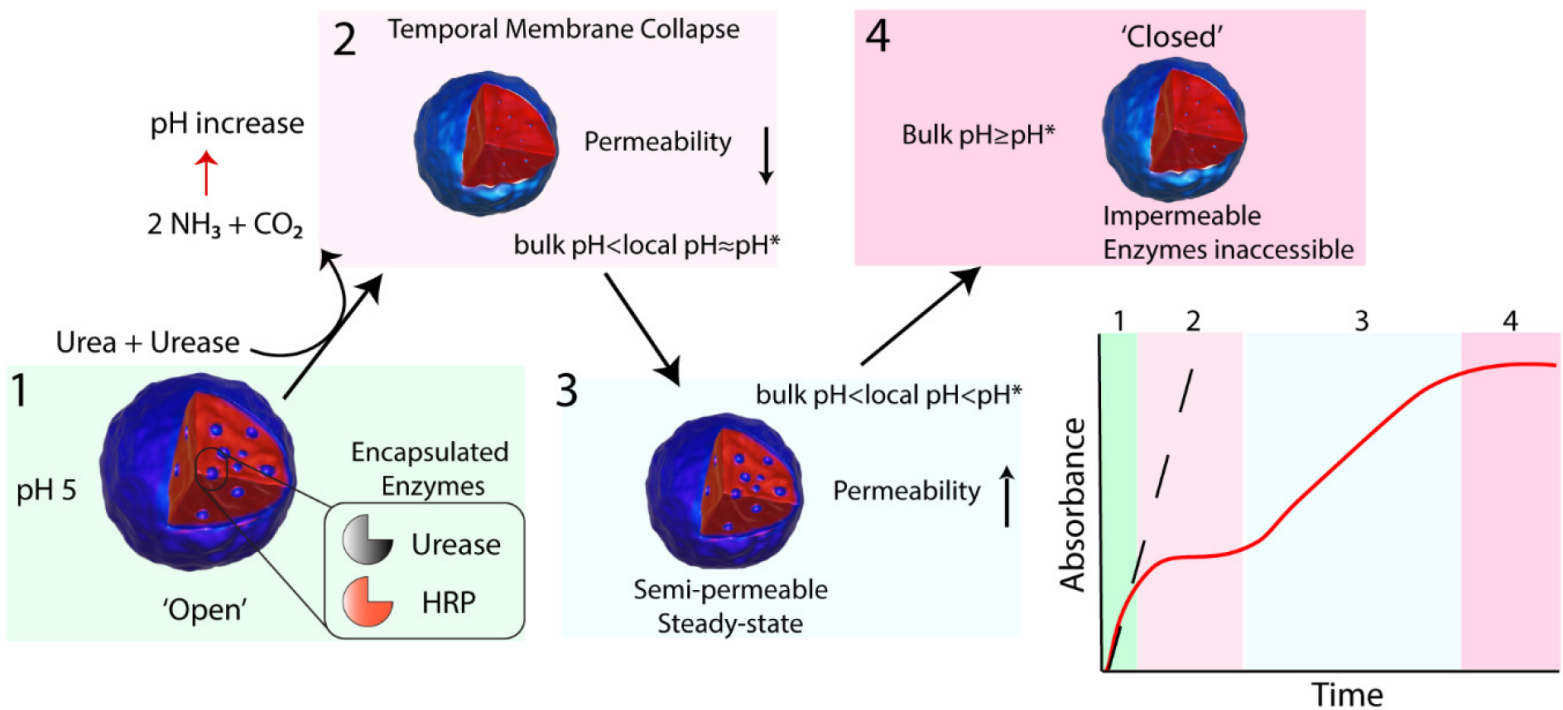
Multiple permeability phases in urease-loaded bicontinuous nanospheres. In stage 1 the nanoreactors are fully permeable in the presence of phosphate buffer (pH 5) and the catalytic output is the highest. Consequently, the significant ammonia production causes deprotonation of the tertiary amines and causes a temporal membrane collapse (phase 2). In phase 3 a semipermeable steady state is established until the bulk pH is sufficiently high by phosphate buffer depletion to cause permanent enzyme deactivation, illustrated by phase 4.

In this work we therefore present the assembly of porous, pH-responsive BCNs loaded with urease and the model enzyme HRP, which exhibit nonlinear permeability and catalytic output (Scheme 1). BCNs are often formulated using complex block copolymer architectures, such as multiarm dendrimers or multiblock polymers.^28,29^ However, it has been reported that simpler diblock polymers are able to form these structures when their hydrophilic fraction is low.^30,31^ We used this to our advantage by increasing the hydrophobic fraction of a block copolymer normally used for the fabrication of pH-responsive polymersomes. One advantage of adapting such a platform is the direct comparison of the transient behavior of BCNs and polymersomes using a very similar chemical composition.

In the case of the BCNs, the transiently regulated catalytic output was characterized by different phases, as illustrated in Scheme 1. In the initial phase (green domain in Scheme 1, numbered 1), the nanoreactors were in their permeable phase, as the reaction was initiated at acidic pH and ABTS conversion happened at the highest rate (bulk pH and local pH ≪ pH*). Urea conversion by urease into ammonia caused the system to progress into phase 2, during which the membrane experienced a temporal collapse. This was caused by the locally produced ammonia, which deprotonated the tertiary ammonium groups of the polymer due to the high local pH even though the bulk pH was significantly lower (bulk pH < pH*, local pH ≈ pH*). This caused HRP to become temporally inaccessible or less accessible for the ABTS substrate, characterized by a plateau or dampening phase. Next, the BCNs progressed to phase 3, during which a steady state was established, and the permeability remained constant, presumably due to balancing proton influx with urea influx and conversion (bulk pH < local pH < pH*). The proton influx from bulk allows for permeabilization, which is accompanied by urea influx and conversion into ammonia. Once the pH was high enough by bulk phosphate buffer depletion, the BCNs became permanently impermeable (phase 4, bulk pH and local pH ≥ pH*).

## Results and Discussion

Polymer building blocks were prepared by atom transfer radical polymerization (ATRP) and were composed of a hydrophilic poly(ethylene glycol) block and a hydrophobic domain comprising pH-responsive 2-(diethylamino)ethyl methacrylate (DEAEMA) and 4-(methacryloyloxy) benzophenone (BMA) as monomers. For BCNs, the block copolymer was designed to have a significantly larger hydrophobic fraction, and its final composition was mPEG_45_-*b*-p[DEAEMA_175_-*g*-BMA_28_]. The hydrophobic block of the polymersomes was shorter, and its composition was mPEG_45_-*b*-p[DEAEMA_100_-*g*-BMA_14_]. This difference in degree of polymerization caused the polymers to assemble into the desired morphologies by the nanoprecipitation method. The assemblies were subsequently cross-linked by light irradiation at λ = 365 nm, at which the benzophenone methacrylate acted as photo-cross-linking initiator, generating alkyl-amino radicals, which resulted in cross-linking by H-abstraction and radical recombination reactions in the hydrophobic domain, arguably also in the hydrophilic PEG domain. This cross-linking prevented dissociation of the nanoreactors at acidic pH and allowed for the reversible swelling and shrinking response. The (dye-labeled) enzymes HRP and urease were encapsulated by mixing them prior to self-assembly using the nanoprecipitation method. The encapsulated enzyme concentration was then determined by UV–vis spectroscopy, and samples were generally diluted to contain ∼12 U/mL for urease and ∼2 U/mL for HRP.

**Figure 1:**
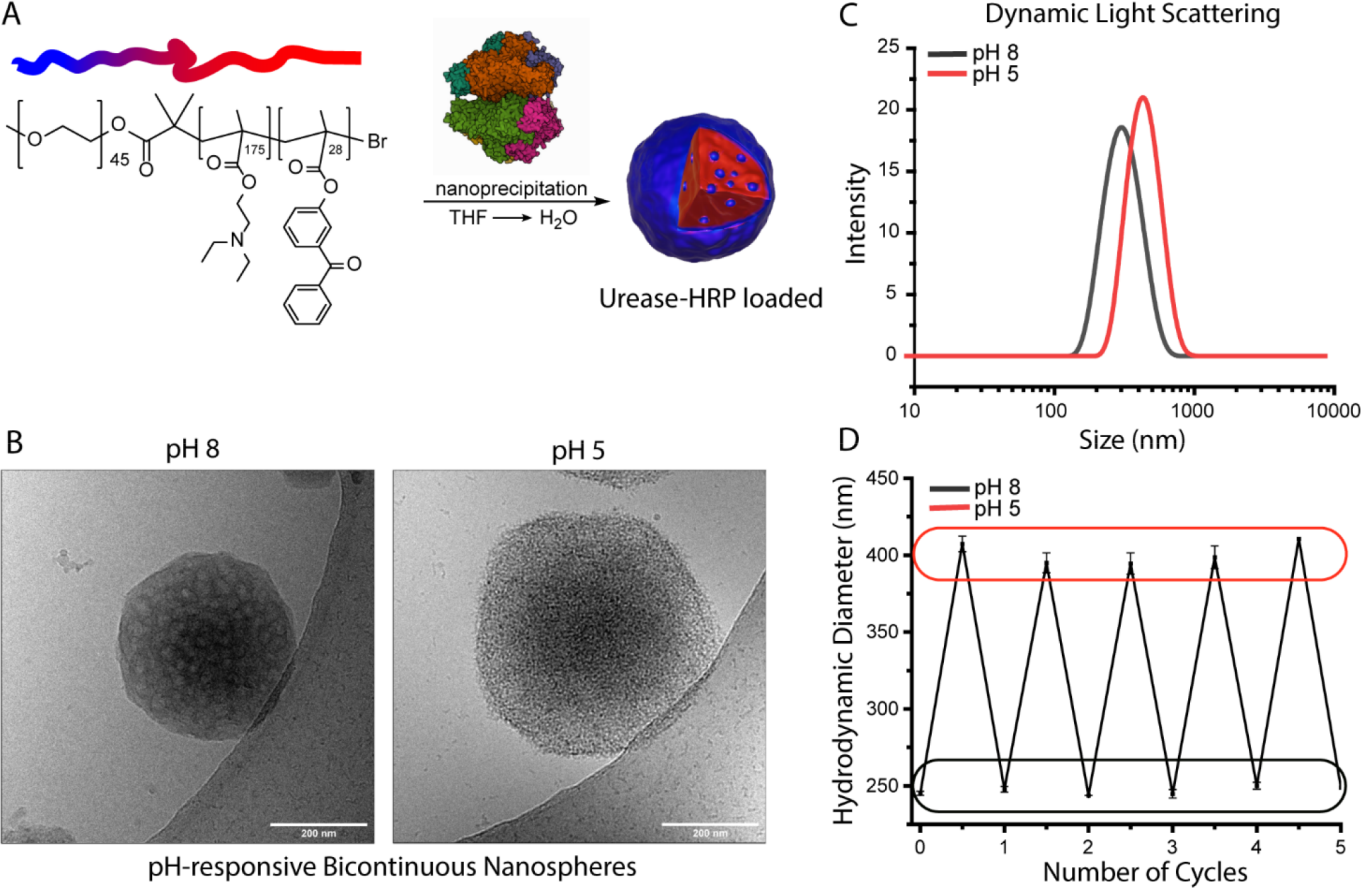
(A) Schematic representation of the preparation of enzyme-loaded bicontinuous nanospheres (BCNs). The polymer (mPEG_45_-*b*-p[DEAEMA_175_-*g*-BMA_28_]) used was synthesized using standard ATRP procedures and consequently assembled into polymeric nanoreactors by means of the nanoprecipitation method. The tertiary amines of DEAEMA introduce pH-responsiveness in the system, while 4-(methacryloyloxy) benzophenone (BMA) is used to cross-link the formed nanoparticles. Cryo-TEM (B) and dynamic light scattering (C, D) show a reversible switch in size by increasing or decreasing the pH. Experimental conditions: 5 mM phosphate buffer.

**Figure 2:**
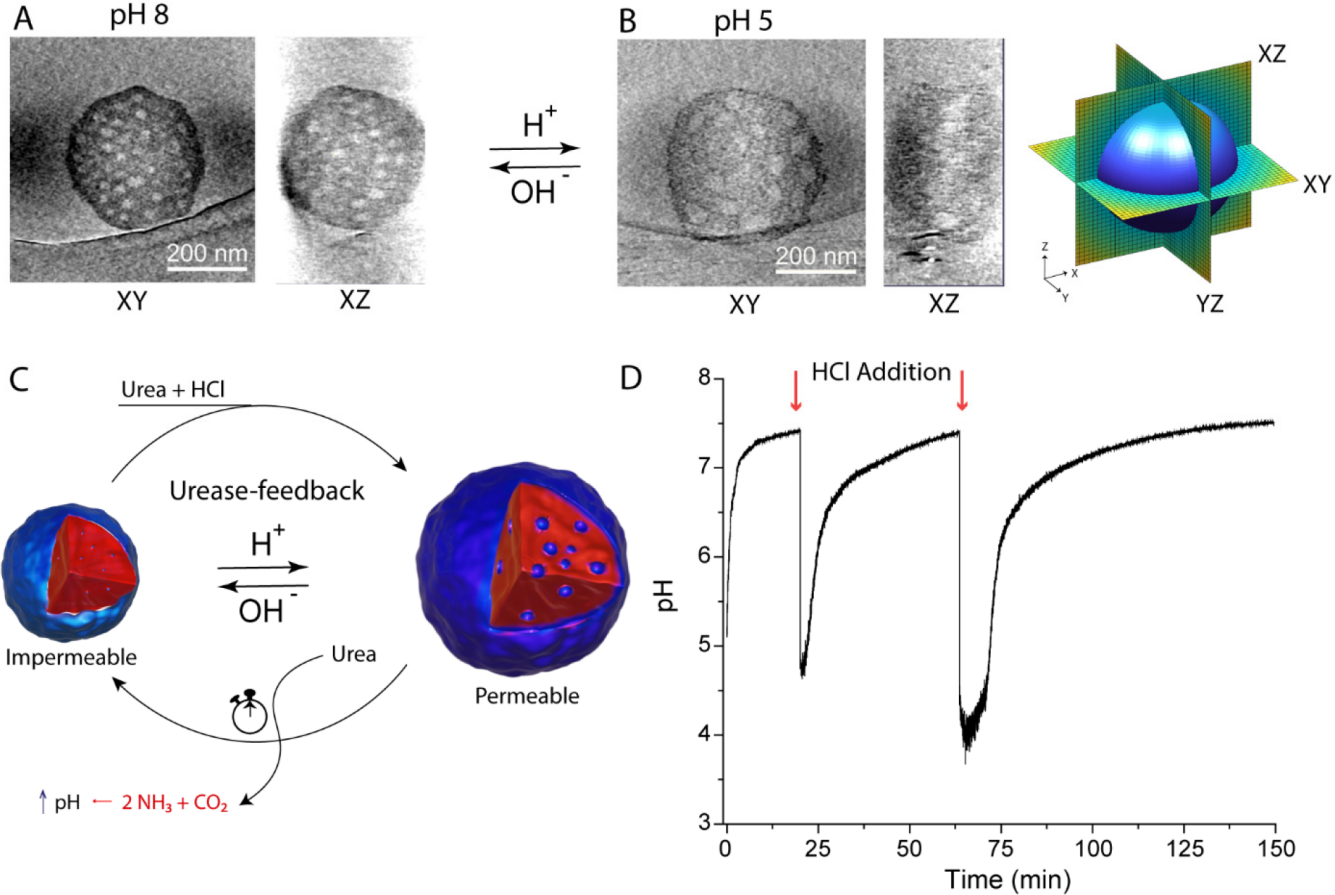
(A) Cryo-electron tomography of pH-responsive BCNs. Cross section (3-frame-averaged) of BCNs at pH 8 in the *xy* and *xz* plane. (B) Cryo-electron tomography cross section (3-frame-averaged) of BCNs at pH 5 in the *xy* and *xz* plane. (C) Schematic illustration of the urea–urease feedback loop: urease is encapsulated in the BCNs. At neutral conditions, nanoreactors are impermeable for the substrate. Acidification of the medium results in a permeability switch, until sufficient urea is converted into ammonia, rendering the BCNs impermeable. (D) Multiple urease feedback loops. Acid is added to restart the feedback loop. Urea is depleted over time (initial concentration 50 mM).

The BCNs were characterized by dynamic light scattering (DLS), cryo-transmission electron microscopy (cryo-TEM), and cryo-electron tomography (Cryo-ET). DLS indicated hydrodynamic radii of 250 nm at pH 8, while the size increased to 400 nm at pH 5 (Figure 1C). This swelling and shrinking behavior was reversible for at least 5 consecutive cycles (Figure 1D). Cryo-TEM confirmed this size change and revealed the complex BCN structure at pH 5 and 8 (Figure 1B). The internal structure of the nanoreactors was further analyzed by cryo-ET, during which tilt series were acquired and a 3D intensity map was reconstructed. Conventional cryo-TEM represents a 2D image of the sample, yet cryo-ET allows for three-dimensional data representation, and cross sections of specific planes are able to be reconstructed, which gives insightful information on the internal structure. Numerical cross sections were extracted from the intensity map to show the internal morphology of the BCN (Figure 2A,B).

The cross-section, obtained from cryo-ET, revealed the existence of well-defined pores, which were not clearly visible using traditional cryo-TEM analysis. In the shrunken, basic conditions, the pores in the XY and XZ plane were small. However, upon acidification, the pore size increased significantly. The initially hydrophobic tertiary amines became hydrophilic upon protonation, causing a considerably larger fraction of the BCN polymer network to become hydrophilic, which resulted in swelling and the formation of larger aqueous pores. This structural analysis gives insight into the mechanism of the permeability changes in the designed BCNs. Additional cryo-TEM data including cross sections and a video of the 3D intensity map from cryo-ET are available in the Supporting Information (Figures S15–S18, SV1 and SV2).

After establishing the pH-dependent morphological changes of the BCNs we next investigated the enzyme-mediated response of these particles in analogy to what we have previously demonstrated for polymersomes. For this purpose, urease-loaded BCNs were exposed to multiple urea–urease feedback loops (Figure 2C), and the pH was measured over time. For this purpose, we utilized a ratiometric dye called C-SNARF-4F,^32^ which enabled a real-time fluorescent readout of the bulk pH by comparing 587/650 nm emissions (Figures S8 and S9). It must be noted that the upper limit of detection of the dye is around pH 8.2. The final pH was validated using a pH-meter. Figure 2D shows that at least 3 cycles were possible, induced by the addition of HCl, whereas no additional urea was added after the initial amount (50 mM) with which the experiment was started. The urea depletion caused consecutive cycles to be slower.

Gumz et al. have shown that increasing the hydrophobic portion shifts the pH at which the turning point for the size transition occurs to a lower pH value.^33^ We defined pH* to be the pH at which the BCNs become impermeable, which was at approximately pH 7.5. This is in good agreement with the size transition measured by DLS following pH titration (Figure S19).

To connect the transient permeability to a pH-independent catalytic process, we coencapsulated HRP inside the BCNs, together with urease. To monitor the HRP activity quantitatively in real time, we used the well-known substrate ABTS, which is converted into ABTS*^+^ in the presence of hydrogen peroxide and is monitored by its characteristic absorbance peak at 415 nm wavelength (Scheme S2, SI). There are multiple ways to probe membrane permeability of nanoreactors, such as dye release assays, PFG NMR spectroscopy, or reaction monitoring.^34^ HRP is widely adopted for the reaction monitoring approach. Since the HRP activity does not change significantly over a wide pH range (Figure S10), the slope of the absorbance–time plots is also a qualitative measure for relative permeability. We utilized this to our advantage and also assessed the relative permeability evolution over time by taking the first derivative of our absorbance–time plots.

**Figure 3:**
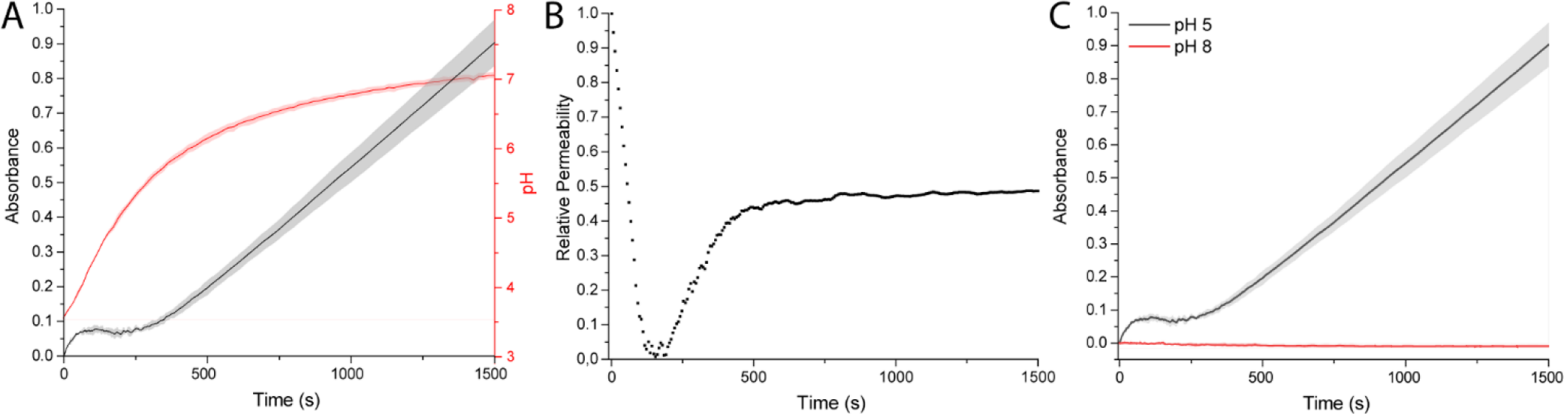
(A) Absorbance–time and pH–time plots of urease–HRP-loaded BCNs. (B) Relative permeability plot of urease–HRP-loaded BCNs. (C) ABTS* production under “open” (pH 5) and “closed” (pH 8) starting conditions. Experimental conditions: 12 U/mL urease and 2 U/mL HRP; 2 mM ABTS, 1 mM H_2_O_2_, 25 mM urea, 5 mM phosphate buffer.

Figure 3A shows the transiently regulated catalytic output of HRP according to the phases mentioned previously in Scheme 1. After the initial phase with rapid catalytic output, the system progressed into phase 2, characterized by the plateau or decrease in ABTS conversion. Consequently, the system stabilized into the steady-state phase, recognizable by the constant catalytic output. A complete urease–HRP BCN cycle is displayed in Figure S23.

This nonlinear behavior was even more evident when the derivative of the ABTS reaction rate was plotted as a measure of relative permeability (Figure 3B), which showed that the permeability reached zero, corresponding to the plateau in Figure 3A, followed by a steady state phase, indicated by a constant permeability. Furthermore, operating under “impermeable” starting conditions (pH 8, pH > pH*) showed no significant ABTS conversion, which demonstrates that at this pH the BCNs were fully impermeable for ABTS (Figure 3C).

**Figure 4:**
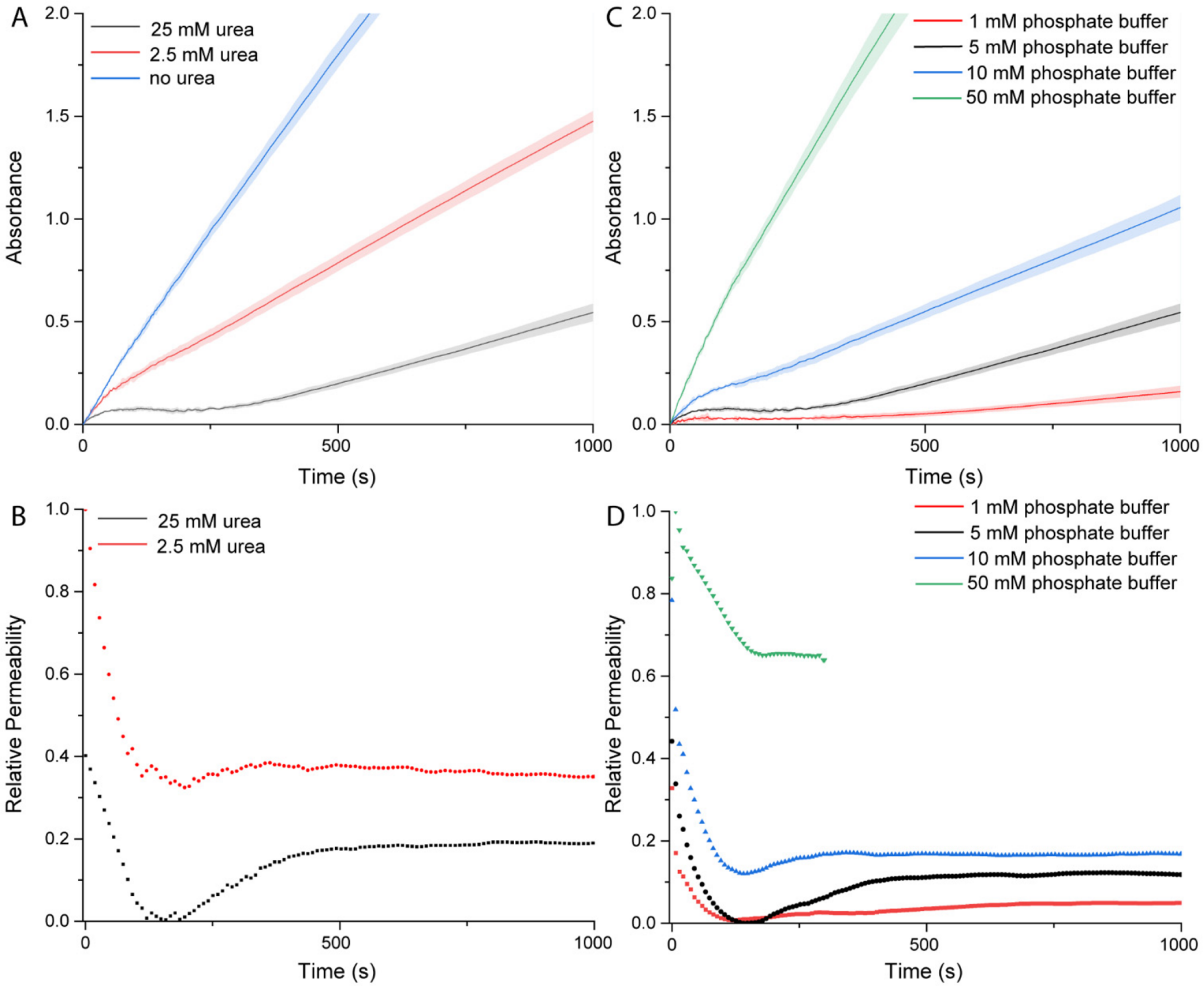
(A) Influence of urea on the nonlinear behavior of urease–HRP-loaded BCNs in 5 mM phosphate buffer. (B) influence of urea on the relative permeability. (C) Influence of buffer capacity on the nonlinear behavior of urease–HRP-loaded BCNs. Urea concentration is 25 mM. (D) Influence of buffer capacity on the relative permeability. For the 50 mM phosphate buffer points were omitted after 300 s due to absorbance exceeding 1.5. Experimental conditions: 12 U/mL urease and 2 U/mL HRP, 2 mM ABTS, 1 mM H_2_O_2_.

To further corroborate the hypothesis of the permeability phases, we investigated the influence of urea and the phosphate buffer capacity. When no urea was added, no dampening region was observed, as illustrated in Figure 4A. In this case, no ammonia was produced, and the pH remained constant, having no effect on the membrane permeability of the BCNs and causing a more standard kinetic profile. In this instance any decrease in activity was caused by substrate depletion. Next, we investigated whether the nonlinear phase could be altered by decreasing the urea concentration from 25 mM to 2.5 mM. At 2.5 mM urea we observed a sharp decrease in enzymatic activity after the initial stage; however, no impermeable phase was reached, as illustrated in Figure 4B. It is likely that the amount of ammonia produced was not sufficient to render the membrane temporally impermeable, and the steady state was achieved more rapidly. Although the membrane did not become impermeable, a small drop in permeability was still observed prior to recovery. Control experiments showed a minimal reduction in HRP activity in the bulk when exposed to 25 mM urea (Figure S11). When the BCNs reached their steady state, the catalytic output of HRP still varied with the amount of urea added. It was tuned from 100% (without urea, control) to 50% (2.5 mM urea) to as low as 25% (25 mM urea) (Figure S12). This phenomenon can be used to temporally regulate catalytic output simply by addition of different amounts of urea.

Similarly, the effect of phosphate buffer capacity was studied. A higher buffer capacity attenuates the effect of the locally produced ammonia, and it was therefore presumed that the BCNs might exhibit a diminished effect on the catalytic output of HRP. Indeed, increasing the buffer capacity from 5 mM to as high as 50 mM prevented the formation of an impermeable phase in the presence of 25 mM urea, as ABTS*^+^ was rapidly produced (Figure 4C,D). In this instance, the phosphate buffer capacity is presumably reasonably high to compensate for the locally produced ammonia. Increasing the buffer capacity from 5 mM to 10 mM still showed significant inhibition and the appearance of the nonlinear regime, albeit less pronounced (Figure 4D). Increasing buffer capacity also increased the steady-state permeability and thus catalytic activity, despite identical substrate concentrations. Applying a buffer capacity of 5 or 1 mM both resulted in the temporal membrane collapse, although the steady-state permeability using 1 mM phosphate buffer was lower. The effect of buffer capacity on the enzymatic activity of HRP in bulk was shown to be negligible (Figure S13). The pH evolution at different buffer capacities was measured and is displayed in Figure S14.

**Figure 5:**
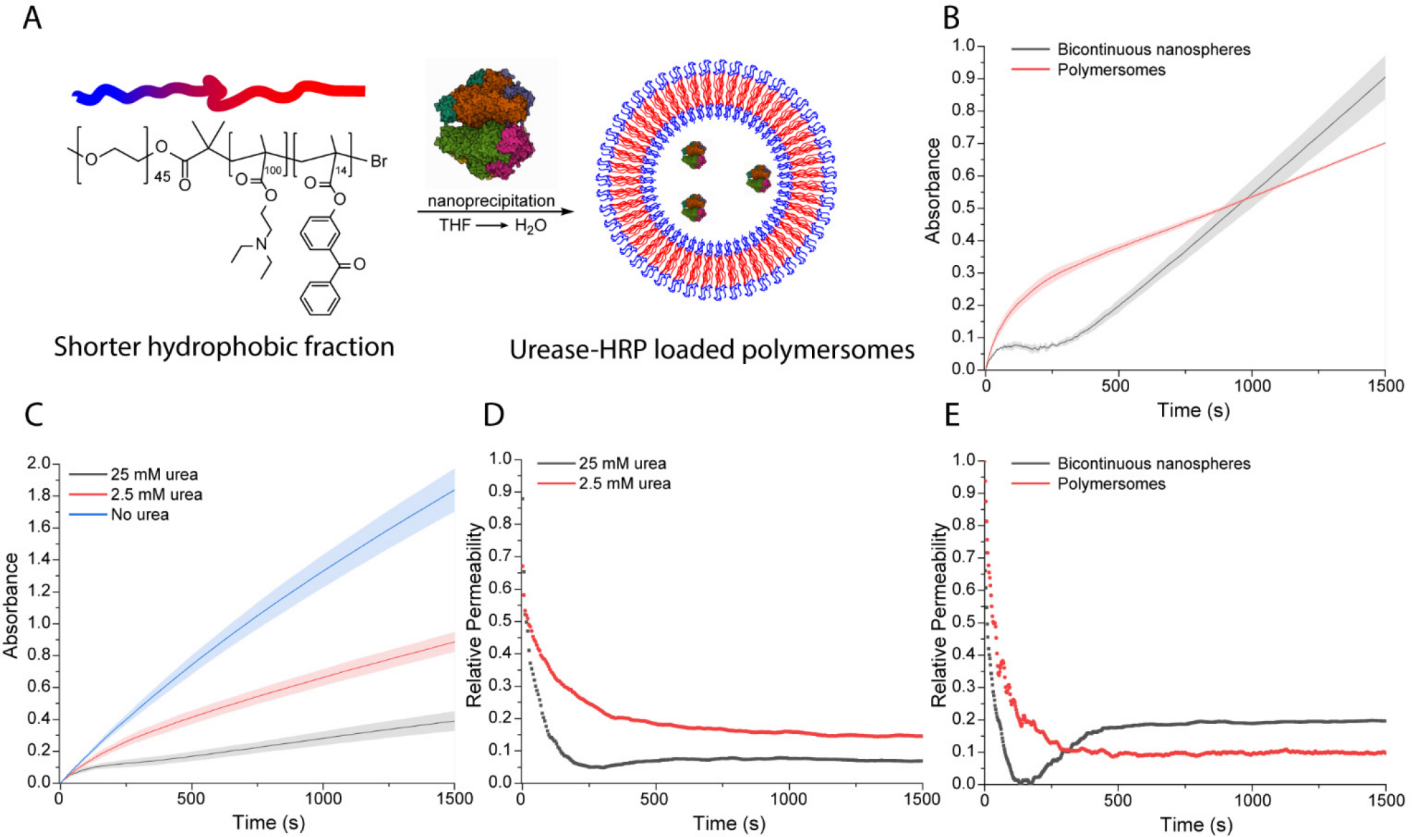
(A) Schematic representation of the preparation of enzyme-loaded polymersomes. Polymer composition for polymersomes is mPEG_45_-*b*-p[DEAEMA_100_-*g*-BMA_14_]. (B) Direct comparison of urease–HRP-loaded polymersomes and urease–HRP-loaded BCNs (12 U/mL urease, ∼2 U/mL HRP, 25 mM urea). (C) Influence of urea on the urease–HRP-loaded polymersomes (6 U/mL urease, 1 U/mL HRP). (D) Influence of urea on the relative permeability of polymersomes. (E) Direct comparison of the relative permeability between BCNs and the polymersomes (derivatives of 5B). All conditions were measured in 5 mM phosphate buffer.

Finally, we compared our BCN system with the well-known pH-responsive polymersome nanoreactors as we hypothesized the dampening region to be nonexistent or less significant in the latter system due to the decreased hydrophobic portion present in the nanoreactors and hence the increased diffusion of substrates and reactants. To this end, a polymer with a shorter hydrophobic portion (mPEG_45_-*b*-p[DEAEMA_100_-*g*-BMA_14_]) was synthesized and self-assembled into polymersomes, coencapsulating both urease and HRP (Figure 5A). Cryo-TEM was performed to confirm the desired morphology (Figure S20), and a bilayer thickness of approximately 20 nm was measured. The catalytic output of the polymersomes was probed by measuring the ABTS conversion, and it became apparent that the polymersomes displayed an HRP activity that was twice as high compared to the BCNs with identical HRP loading, indicating that the intrinsic permeability of the polymersomes is higher (Figure S21).

Next, it was investigated whether the polymersomes also exhibited nonlinear phases under the influence of 25 mM urea, and the direct comparison with the BCNs was visualized in Figure 5B. It became apparent that the polymersomes also experienced a decrease in catalytic output when urea was present. Next, the urea concentration was decreased to 2.5 mM, and a control without the addition of urea was measured (Figure 5C). Similar to the BCNs, urea inhibited the catalytic output of HRP by reducing the membrane permeability as a response to locally produced ammonia. However, the permeability followed a different trajectory and decreased more gradually over time, as illustrated in Figure 5D. The bulk pH evolution over time of the urease–HRP-loaded polymersomes is displayed in Figure S22.

This difference in permeability profile emphasizes the effect of the morphology on the transient behavior of these nanoreactors, which is illustrated by the direct comparison of the permeability in Figure 5E.

Overall, the nonlinear permeability phases occur due to encapsulation of a base-producing enzyme. Furthermore, the morphology and more specifically the membrane structure of our pH-responsive nanoreactors strongly dictate the response and permeability profile.

## Conclusion

In this work we presented the synthesis and assembly of porous, pH-responsive bicontinuous nanospheres whose permeability can be switched “ON” by acidification and autonomously be turned “OFF” by employing the urea–urease feedback loop, which produces ammonia and in turn increases the pH over time. The nanoreactor morphology was validated by cryo-electron tomography, which also verified the change in BCN pore size upon changes in pH. This transient permeability was extended to the transient activation of enzymatic activity of HRP. When BCNs loaded with both urease and HRP are exposed to low pH, their structure is swollen and the enzyme accessible for the substrate. The production of basic ammonia by urease however quickly increases the local pH, which strongly decreases the permeability and slows down the catalytic conversion, causing a nonlinear permeability profile. Decreasing the urea concentration reduces the decrease in permeability. Similarly, an increase in phosphate buffer capacity prevents the formation of the impermeable phase and diminishes the effect of locally produced ammonia.

This unique nonlinear permeability profile is only clearly observable for BCNs and not for polymersomes, due to the presence of an increased hydrophobic barrier in the former topology that contributes to an increase in the responsiveness.

As far as we know, this nonlinear adaptive behavior has not been observed before in nanoreactor systems. This concept could be applicable to a larger class of pH-responsive nano- or even microreactors, in which the local control of pH could also be used to create a unique microenvironment for catalytic processes.

## Supporting Information Available

Experimental details, supporting figures, and characterization data (PDF)
Video SV1: 3D intensity map from cryo-ET (MP4)
Video SV2: 3D intensity map from cryo-ET (MP4)

## ABBREVIATIONS

BCN: bicontinuous nanosphere
HRP: horseradish peroxidase
DEAEMA: 2-(diethylamino)ethyl methacrylate
BMA: benzophenone-methacrylate
PEG: poly(ethylene glycol)
DLS: dynamic light scattering
cryo-TEM: cryo-transmission electron microscopy
cryo-ET: cryo-electron tomography
ABTS: 2,2′-azino-bis(3-ethylbenzothiazoline-6-sulfonic acid) diammonium salt;
