# Supplementary material for: Nonlinear Transient Permeability in pH-Responsive Bicontinuous Nanospheres
Source: J Am Chem Soc. 2023 Mar 30;145(15):8600–8. doi: 10.1021/jacs.3c01203 (PMC10119974; doi:10.1021/jacs.3c01203)
Supplement: Supplementary file 1 — ja3c01203_si_001.pdf [file ja3c01203_si_001.pdf]

Non-linear Transient Permeability in pH-responsive Bicontinuous Nanospheres

Wouter P. van den Akker[1,2], Hanglong Wu[1], Pascal L.W. Welzen[1], Heiner Friedrich[3], Loai K.E.A. Abdelmohsen[1], Rolf A.T.M. van Benthem[3,4], Ilja K. Voets[2] and Jan C.M. van Hest*[1]

Supplementary Information

[1]Department of Chemistry & Chemical Engineering, Institute for Complex Molecular Systems, Bio-Organic Chemistry, Eindhoven University of Technology, Helix, P.O. Box 513, 5600MB Eindhoven (The Netherlands)

[2]Department of Chemistry & Chemical Engineering, Self-Organizing Soft Matter
Eindhoven University of Technology, P.O. Box 513, 5600MB Eindhoven (The Netherlands)

[3]Department of Chemistry & Chemical Engineering, Laboratory of Physical Chemistry and Center for Multiscale Electron Microscopy. Eindhoven University of Technology, 5600MB Eindhoven (The Netherlands)

[4]Energy Transition Center Amsterdam
Grasweg 31, 1031 HW Amsterdam (The Netherlands)

Corresponding Author
j.c.m.vanhest@tue.nl

1. Materials

All materials were used as received unless stated otherwise.

Horseradish peroxidase from *Amoracia rusticana* (Type VI, 295 U/mg) and urease from *Canavalia ensiformis* (Type IX, 72.5 U/mg) were purchased from Sigma Aldrich. 2-(Diethylamino)ethyl methacrylate (99%), poly(ethylene glycol) methyl ether 2-bromoisobutyrate (Mw/Mn 1.07), ethylene glycol dimethacrylate (98%), poly(ethylene glycol) methacrylate, 2-hydroxyethylmethacrylate (98%), Urea (≥98%), 2,2′-azino-bis(3-ethylbenzothiazoline-6-sulfonic acid) diammonium salt (98%), Rhodamine B isothiocyanate were purchased from Sigma Aldrich. 4-Methacryloyloxy benzophenone was purchased from TCI chemicals. SNARF™-4F 5-(and-6)-carboxylic acid (96%) and Alexa Fluor 647 NHS-ester (99%) were purchased from Thermo Fisher. 2-(Diethylamino)ethyl methacrylate was passed through an alumina column to remove inhibitor.

2. Instruments

Nuclear Magnetic Resonance Spectroscopy (NMR). ^{1}H NMR spectra were recorded on a Bruker (400MHz) spectrometer with $CDCl_3$ as solvent.

Size-Exclusion Chromatography (SEC). The dispersities of the polymers were measured using a Shimadzu Prominence-I SEC system with a PL gel 5 µm mixed D and mixed C column (Polymer Laboratories) calibrated with PS standards and equipped with a Shimadzu RID-20A differential refractive index detector. THF was used as an eluent with a flow rate of 1 mL/min

Dynamic Light Scattering (DLS). DLS measurements were performed on a Malvern Zetasizer Nano ZSP at room temperature. All measurements were done in triplicates.

UV-Vis Spectroscopy (UV-Vis). Time-dependent UV-Vis measurements were performed on a Cary 3500 UV-Vis spectrophotometer. Measurements were done approximately once every 0.6 seconds with an averaging time of 0.1 s. A quartz cuvette with a pathlength of 1 cm was used.

Plate reader. Time-dependent absorbance and fluorescence readings were measured on a Tecan™ Spark 10M plate reader using Corning Falcon 96 Black Flat Transparent well plates. Tecan™ Spark 10M has linear OD measurements between 0-4 OD.

UV Photoreactor. The crosslinking of the nanoreactors was performed using a Luzchem **LZC-4V** photoreactor equipped with 14 UVA lamps with a wavelength of 365 nm and power density of 5 mW/cm^2.

Cryogenic transmission electron microscopy (cryo-TEM) and cryo-electron tomography (cryo-ET):

Cryo-TEM and cryo-ET were performed on the TU/e CryoTITAN (Thermo Fisher Scientific) equipped with a field-emission gun operating at 300 kV, an autoloader station and a post-column Gatan energy filter. The TEM grids (R2/2, Cu, Quantifoil Jena grids, Quantifoil Micro Tools GmbH) were firstly plasma treated in a Cressington 208 carbon coater for 40 seconds before being used. Then, a 3 μL nanoparticle solution was pipetted onto the grid and blotted in a Vitrobot MARK IV (Thermo Fisher Scientific) at 100% humidity. Note that for cryo-ET sample preparation, 10 nm gold nanoparticles (nanoComposix, Inc), which were used as fiducial markers, were added into an aliquot of the dispersion before vitrification. No morphological changes were found on both samples at pH 5 and pH 8, indicating the introduction of the gold nanoparticle dispersion didn't significantly affect the pH of our sample solution. The grid was then blotted for 3 seconds (blotting force $^{-}3$) and directly plunged and frozen in liquid ethane. Images were acquired via a post-GIF 2k Gatan CCD (charge-coupled device) camera.

Cryo-ET tilt-series acquisition was carried out using Inspect 3D software (Thermo Fisher Scientific). Dose series were firstly acquired up to ~ 90 $e^{-}\cdot Å^{-2}$ on both samples at pH 5 and pH 8 before recording the tomogram, showing no changes on the sample morphology. Therefore, an optimal electron flux of 2.6 $e^{-}\cdot Å^{-2}\cdot s^{-1}$ was used with an exposure time of 0.6 s/frame, resulting in a total dose of ~ 72 $e^{-}\cdot Å^{-2}$. Alignment of the tilt-series and tomographic reconstruction were performed in IMOD using the simultaneous iterative reconstruction technique (SIRT, 20 iterations). The parameters using in cryo-ET are shown below:

Angular sampling: $^{-}68°$ to 68° at 3° increments;

Magnification: 15000 ×; Defocus: $^{-}5$ μm;

Total image number: 46; Total electron dose: ~ 1.56 $e^{-}.Å^{-2}$ /frame;

3. Methods

3.1.1

Synthesis of amphiphilic poly[ethylene glycol]-b-poly[diethylaminoethyl methacrylate-g-benzophenone methacrylate] mPEG$_{45}$-b-p[DEAEMA$_{175}$-g-BMA$_{28}$] - Composition for formation of bicontinuous nanospheres

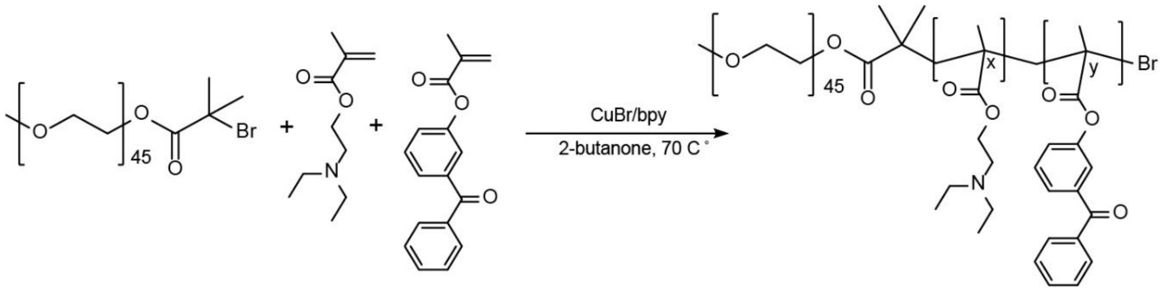

Scheme S1. Synthesis of mPEG-b-p[DEAEMA-g-BMA] by ATRP

mPEG- macroinitiator (0.05 mmol, 100 mg), diethylaminoethyl methacrylate (DEAEMA) (10 mmol, 2 mL) and benzophenone methacrylate (BMA) (1.12 mmol, 300 mg) were added to a Schlenk flask and dissolved in 6 ml 2-butanone. Subsequently, 2,2'-bipyridine (0.1 mmol, ~16 mg) was added to the solution. The CuBr (0.1 mmol) catalyst was added and the flask was immediately frozen in liquid N_2 and subjected to three freeze-pump-thaw cycles before placing it in a preheated oil bath at 60 °C. After the reaction, the solution was diluted with 60 mL THF and passed through an alumina column to remove the catalyst. The filtrate was concentrated and precipitated in cold hexane to yield the final polymer.

^{1}H NMR (399 MHz, $CDCl_3$): δ = 1.03 (s), 2.57 (m), 2.69 (s), 3.64 (s), 4.00 (s), 7.15-7.9 (m)
The degree of polymerization was determined by ^{1}H NMR with the PEG45 macroinitiator as reference (180 protons)

GPC : **Đ = 1.53**

3.1.2

Synthesis of amphiphilic poly[ethylene glycol]-b-poly[diethylaminoethyl methacrylate-g-benzophenone methacrylate] (mPEG$_{45}$-b-p[DEAEMA$_{100}$-g-BMA$_{14}$] – Composition for formation of polymersomes

mPEG-macroinitiator (0.05 mmol, 100 mg), DEAEMA (1.25 mmol, 1.6 mL) and BMA (0.75 mmol, 200 mg) were added to a Schlenk flask and dissolved in 5 ml 2-butanone. Subsequently, 2,2'-bipyridine (0.1 mmol, ~16 mg) was added to the solution. The CuBr catalyst was added and the flask was immediately frozen in liquid N_2 and subjected to three freeze-pump-thaw cycles before placing it in a preheated oil bath at 60 °C.
After the reaction, the solution was diluted with 60 mL THF and passed through an alumina column to remove the catalyst. The filtrate was concentrated and precipitated in cold hexane to yield the final polymer.

^{1}H NMR (399 MHz, $CDCl_3$): δ = 1.03 (s), 2.57 (m), 2.69 (s), 3.64 (s), 4.00 (s), 7.15-7.9 (m)
The degree of polymerization was determined by ^{1}H NMR with the PEG45 macroinitiator as reference (180 protons)

GPC : **Đ = 1.37**

Note that for the formation of polymersomes a polymer with a smaller hydrophobic fraction is required, and smaller amounts of DEAEMA & BMA are added during the polymerization.

3.2.1 Preparation of Rhodamine-B labeled urease (RhB-urease)

Urease was labeled with Rhodamine-B to determine the enzyme concentration inside the nanoreactors. Rhodamine-B isothiocyanate was dissolved in DMSO to obtain a stock solution of 10 mg/ml. Next, 20 µL of this solution was added to 2.5 mL 30 mg/ml urease dissolved in 5 mM phosphate buffer at pH 8. The reaction was stirred for 5 hours at room temperature. Subsequently, the RhB-urease was transferred to a 12-14 kDa MWCO dialysis membrane and dialyzed against 5 mM phosphate buffer (pH 8) to remove unreacted RhB-isothiocyanate. The phosphate buffer was refreshed twice.

The degree of labeling (DOL) was determined by the following formula :

$$[DOL] = \frac{A_{RhB}}{\varepsilon_{RhB} \cdot [Urease]}$$

The DOL was calculated to be 0.46, indicating an average number of 0.46 RhB conjugated per urease. The UV/Vis spectrum of RhB-urease and the corresponding calibration curve are displayed in figure S5.

3.2.2 Preparation of Alexa Fluor 647 labeled Horse Radish Peroxidase (AF647-HRP)

Horse Radish Peroxidase was labeled with a red-shifted dye, AlexaFluor 647 (AF647) to determine the absorbance more accurately. AlexaFluor 647 NHS-ester was dissolved in DMSO to obtain a stock solution of 1 mg/ml. Next, 60 µL AF647 was added to 2 mL 2 mg/ml HRP dissolved in 5 mM phosphate buffer at pH 8. The reaction was stirred for 5 hours at room temperature. Subsequently, the AF647-HRP was transferred to a 12-14 kDa MWCO dialysis membrane and dialyzed against 5 mM phosphate buffer (pH 8) to remove unreacted AlexaFluor 647 NHS-ester. The buffer was refreshed twice during dialysis. The degree of labeling (DOL) was determined by the following formula:

$$[HRP] = \frac{A_{403}}{\varepsilon_{403}}$$

Where A_{403} is the characteristic absorbance band of horse radish peroxidase (ε = 102000 M^{-1} cm^{-1}).

$$[DOL] = \frac{A_{AF647}}{\varepsilon_{AF647} \cdot [HRP]}$$

The DOL was calculated to be 0.5, indicating an average number of 0.5 AF647 conjugated per HRP. The UV/Vis spectrum of AF647-HRP and the corresponding calibration curve are displayed in figure S6.

3.3 Formation of polymeric nanoreactors

15 mg RhB-urease and/or 1 mg AF647-HRP were dissolved in 1 ml 5 mM phosphate buffer (pH 7.4) in a 4 mL vial equipped with a magnetic stirrer rotating at 450 rpm. The block copolymer mPEG-b-p(DEAEMA-g-BMA) was dissolved in 1 ml THF to obtain a final concentration of 5 mg/ml. 1 mL polymer solution was added to 1 mL enzyme solution with a flowrate of 1 mL/h via a syringe pump. In case of the bicontinuous nanospheres, the cloudy suspension was immediately crosslinked by UV irradiation and transferred to a 12-14 kDa MWCO membrane and dialyzed against 5 mM phosphate buffer (pH 7.4), while occasionally refreshing the phosphate buffer. For the polymersome formation, the assembly was crosslinked after the first dialysis step. Next, the formed nanoreactors were transferred to a 1 MDa Float-a-lyzer and dialyzed against 5 mM phosphate buffer overnight to remove non-encapsulated enzymes. Finally, the nanoparticles were centrifuged for 5 minutes at 4000 rpm via spin filtration over 0.1 µm membranes. The filtrate was analyzed for dye absorbance to check whether any non-encapsulated enzymes were still present. In that case, the nanoreactors were redispersed in 1 mL 5 mM phosphate and subjected to another spin filtration cycle. It must be noted that some particles stick to the filter and are unable to be redispersed, resulting in loss of product.

The enzyme concentration inside the polymersomes and BCNs was determined by UV/Vis spectroscopy by measuring the RhB-urease and AF647-HRP absorbance. 50v% DMSO was added to the polymeric nanoreactor to destroy the structure, releasing the enzymes. Figure S7 shows the UV/Vis spectrum. The amount of encapsulated enzyme was calculated to be around 1.66 mg/ml (~120 U/ml) for RhB-urease and 68 µg/ml (~20 U/ml) for HRP. This translates to an encapsulation efficiency of about 11% for RhB-urease and 6.8% for AF647-HRP. It must be noted that during the purification of the enzyme-loaded nanoreactors some product is lost and real encapsulation efficiencies could be higher.

3.4 Calibration curve of ratiometric C-SNARF-4F dye

1 mg C-SNARF-4F was dissolved in 1 mL DMSO to obtain a stock solution of 1 mg/ml. Multiple samples were prepared at different pHs with a final dye concentration of 0.01 mg/ml in 5 mM phosphate buffer. The fluorescence at 587 nm and 650 nm emission wavelength was determined by a plate reader assay with an excitation wavelength of 525 nm. The raw spectra and the calibration curves are depicted in figures S8 & S9.

3.5 Photocrosslinking of polymersomes & bicontinuous nanospheres.

For nanoreactor crosslinking, the formed nanoreactors were placed in a UV oven and irradiated (365 nm) for 5 minutes at a light intensity of 5 mW/cm^2.

3.6 HRP activity assays

The influence of urea, phosphate buffer capacity and pH on the HRP activity was investigated. 0.1 µg/ml of HRP in 5 mM phosphate buffer (pH 7) was used for the urea and pH studies, during which the urea concentration was varied from 1 to 2.5 and 25 mM urea. The conversion of ABTS was followed over time by following the 415 nm absorbance using UV-Vis spectroscopy or plate reader and the slope was calculated as representative of the relative enzymatic activity between respective conditions.

The effect of pH was studied by varying the pH from 5 to 7 with 0.5 pH increments. (5 mM phosphate).

The effect of buffer capacity was studied by varying the phosphate buffer concentration from 5, 10 to 50 mM phosphate (pH 7). The results are illustrated in figures S10, S11 and S13.

The concentrations of ABTS and H_2O_2 were kept constant at values of 2 mM and 1 mM respectively.

3.7 Urease-loaded BCNs feedback loop cycles

250 µL of urease-loaded BCNs was mixed with 250 µL substrate solution (100 mM urea, 5 mM phosphate, 0.01 mg/ml C-SNARF-4F ratiometric pH dye) in a cuvette suitable for fluorescence. The 587 & 650 nm fluorescence were measured approximately every second using a fluorescence spectrophotometer. At the end of the enzymatic cycle, 2 µL of 0.1M HCl was added to restart the cycle.

3.8 Enzymatic assays of HRP-urease loaded BCNs & polymersomes.

250 µL of enzyme-loaded polymersome/BCN solution was mixed with 250 µL substrate solution in a quartz cuvette. The absorbance was measured approximately once every 0.6 seconds.
For the urea studies, the urea concentrations were 25 mM, 2.5 mM urea and a control without urea. For the buffer capacity studies, the phosphate buffer concentration was varied from 1 mM, 5 mM, 10 mM to 50 mM phosphate. Both the nanoreactor and the substrate solution had the same phosphate buffer concentration. Enzyme concentrations were typically 12 U/ml for urease and 2 U/ml for HRP unless stated otherwise.

ABTS and H_2O_2 concentrations were kept constant during the studies. The substrate ABTS had a final concentration of 2 mM and H_2O_2 of 1 mM.

4. Supplementary Figures

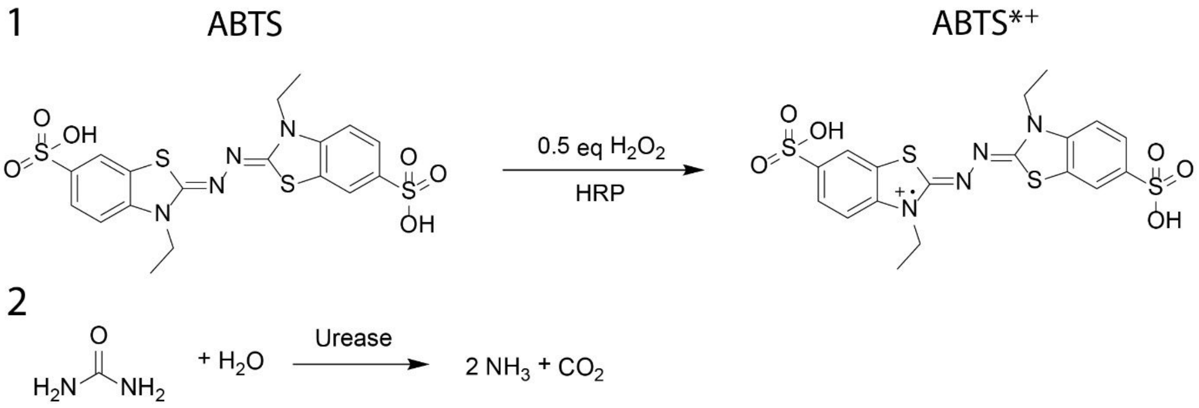

Scheme S2: (1) Enzymatic conversion of 2,2′-azino-bis(3-ethylbenzothiazoline-6-sulfonic acid) to $ABTS^{*+}$ by horse radish peroxidase. (2) Enzymatic conversion of urea into ammonia by urease.

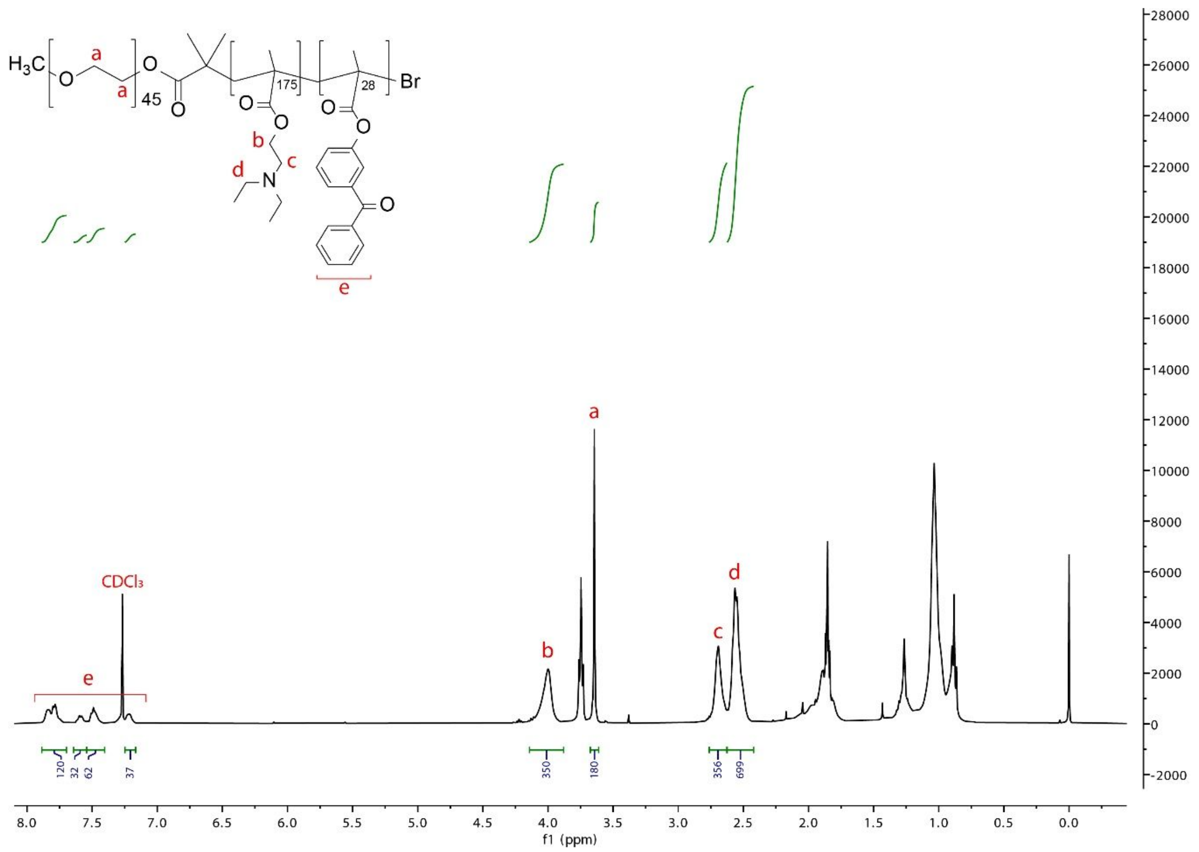

Figure S1. ^{1}H NMR spectrum of **mPEG$_{45}$-b-p[DEAEMA$_{175}$-g-BMA$_{28}$]**

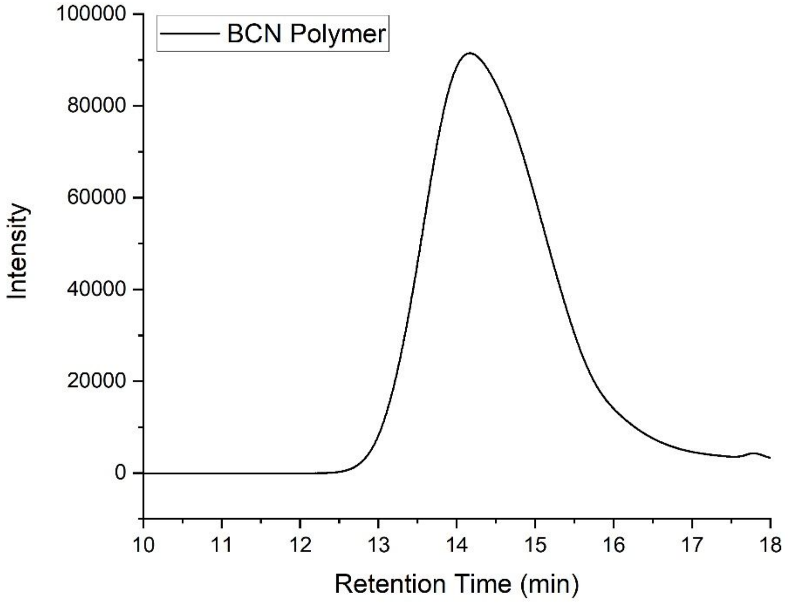

Figure S2. GPC spectrum of **mPEG$_{45}$-b-p[DEAEMA$_{175}$-g-BMA$_{28}$] Đ = 1.53**

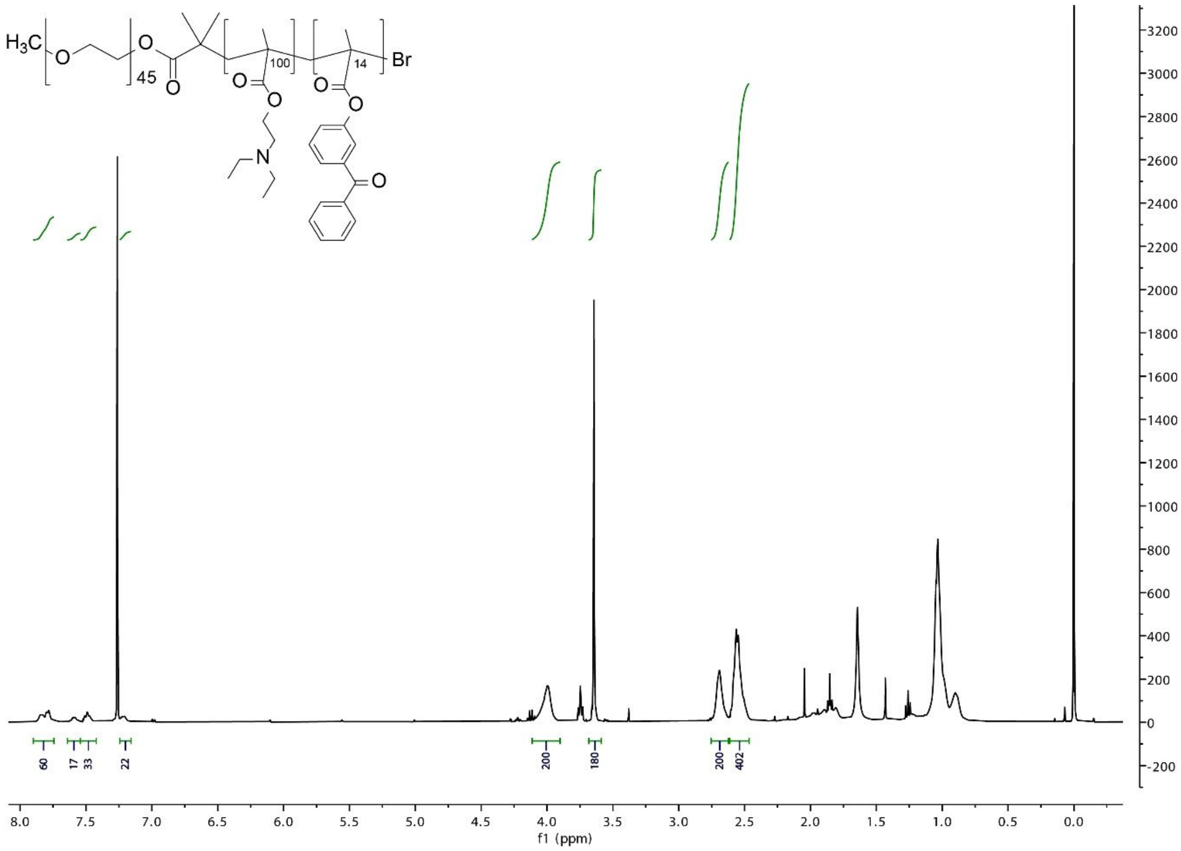

Figure S3. ^{1}H NMR spectrum of **mPEG$_{45}$-b-p[DEAEMA$_{100}$-g-BMA$_{14}$]**

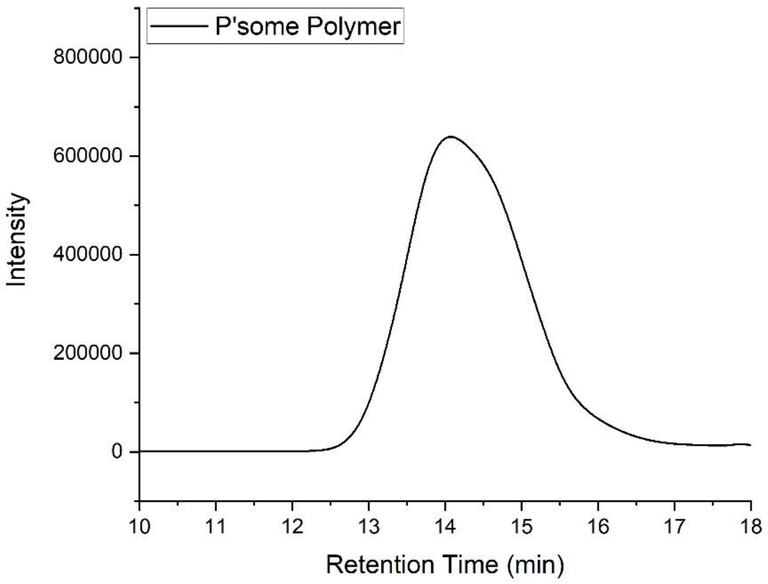

Figure S4. GPC spectrum of **mPEG$_{45}$-b-p[DEAEMA$_{100}$-g-BMA$_{14}$] Đ = 1.37**

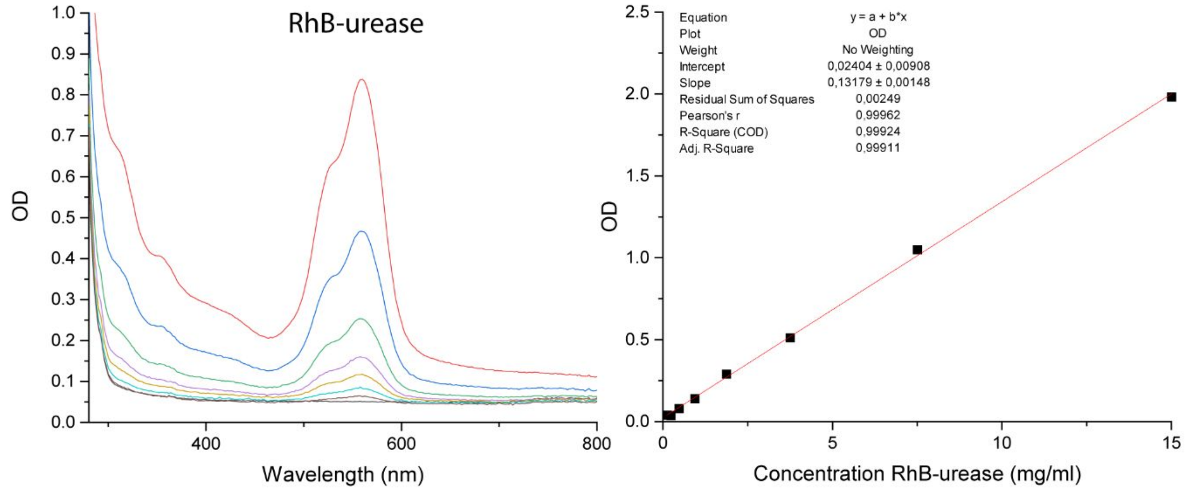

Figure S5. Absorbance spectrum of Rhodamine B-urease conjugate & calibration curve (558 nm wavelength) Note: Absorbance scan is not pathlength adjusted, OD data for the calibration curve is pathlength adjusted to 1 cm pathlength.

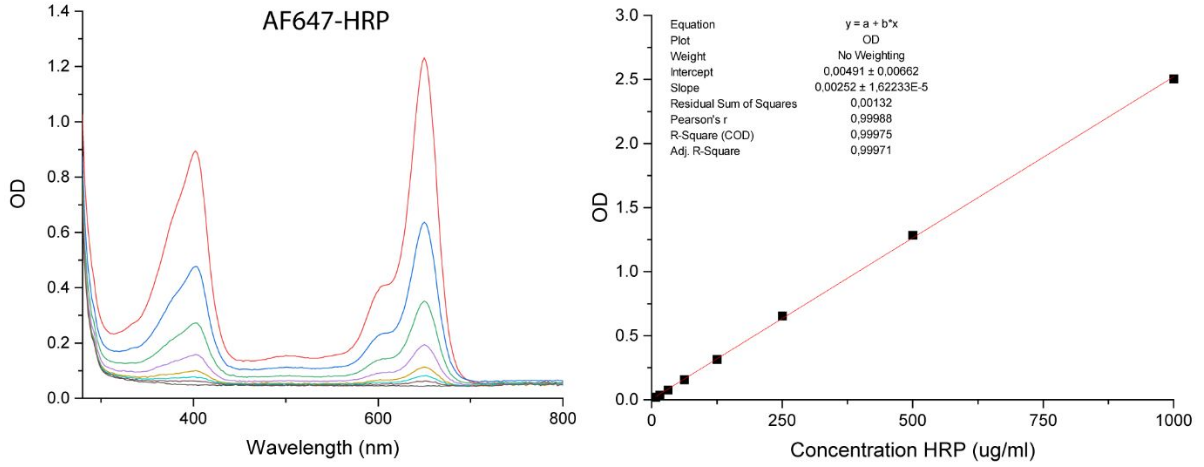

Figure S6. Absorbance spectrum of AlexaFluor647-horse radish peroxidase conjugate (650 nm wavelength) Note: Absorbance scan is not pathlength adjusted, OD data for the calibration curve is pathlength adjusted to 1 cm pathlength.

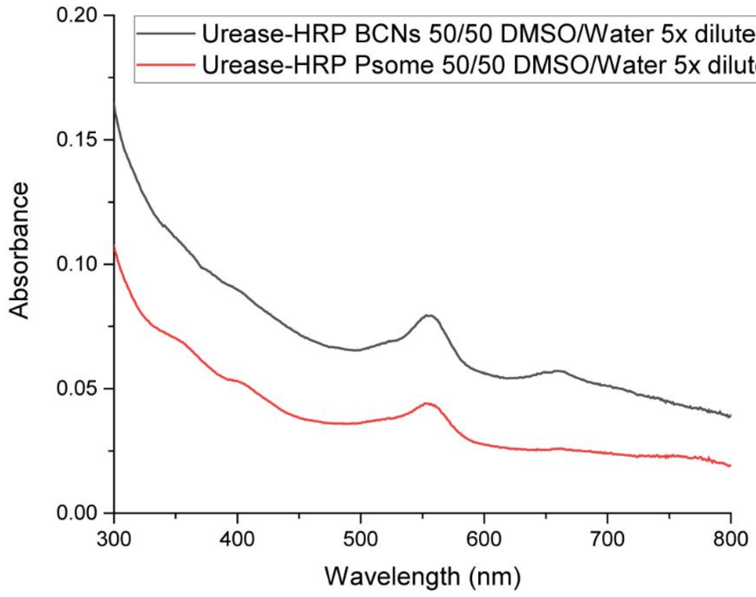

Figure S7. Absorbance spectrum of enzyme loaded BCNs (black curve) and polymersomes (red curve).

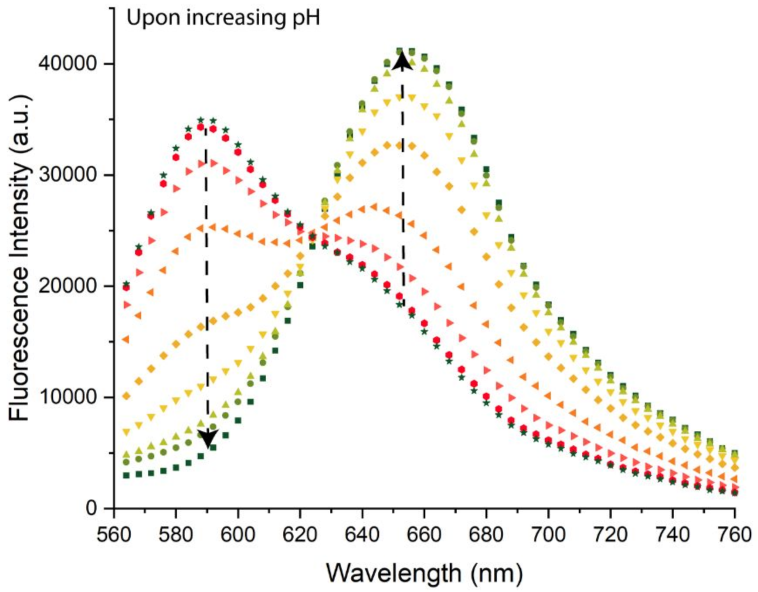

Figure S8. C-SNARF-4F ratiometric dye pH series. The 587 nm fluorescence decreases upon increasing the pH while the 650 nm fluorescence increases.

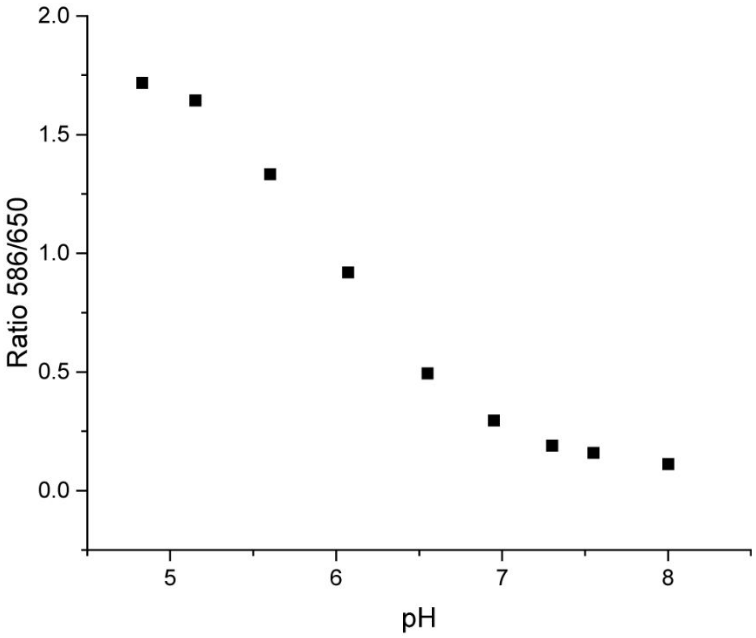

Figure S9. C-SNARF-4F ratiometric dye calibration curve.

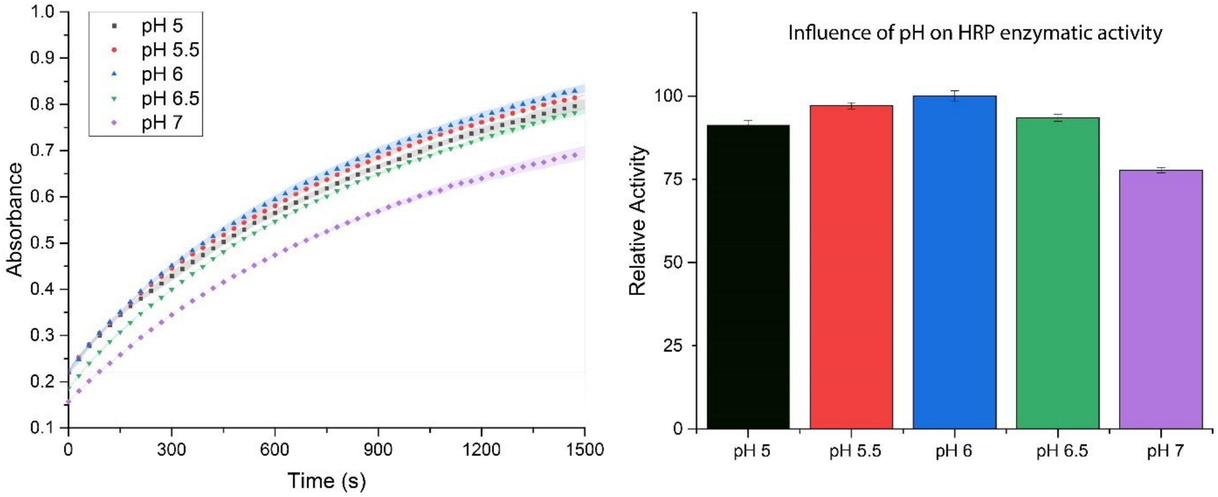

Figure S10. Influence of pH on the HRP activity. The slopes were determined for the first 150 seconds in the linear regime, and the relative activities were calculated. The most active condition was normalized as 100% relative activity (pH 6 condition). The HRP activity does not change significantly at the varied pH.

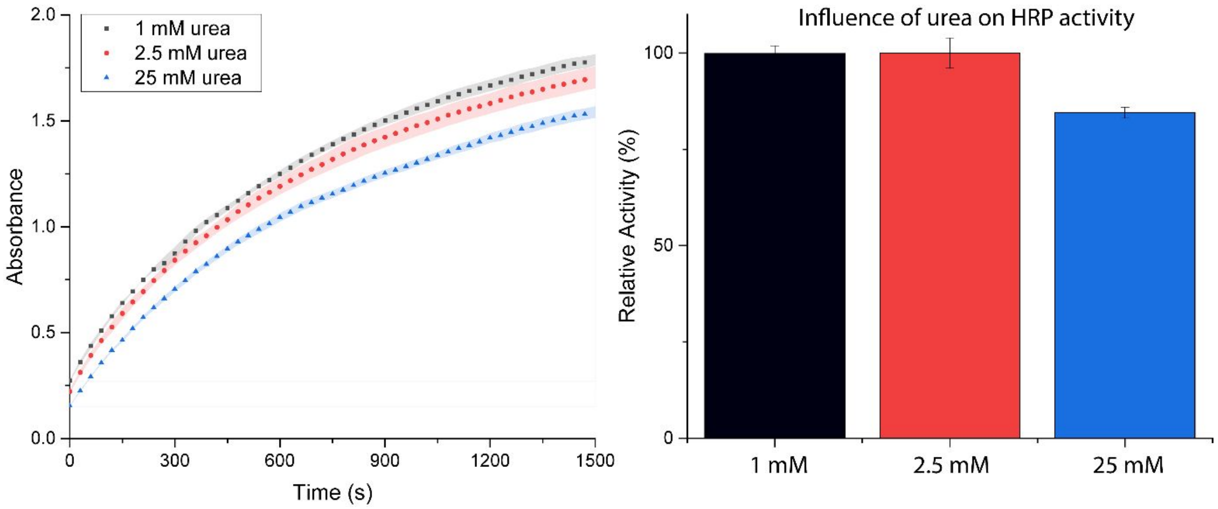

Figure S11. Influence of different urea concentrations on the HRP activity. The slopes were determined for the first 150 seconds in the linear regime, and the relative activities were calculated. Urea concentrations were varied from 1 to 25 mM. The relative activities show that higher urea concentrations cause a small decrease in the enzymatic activity of HRP.

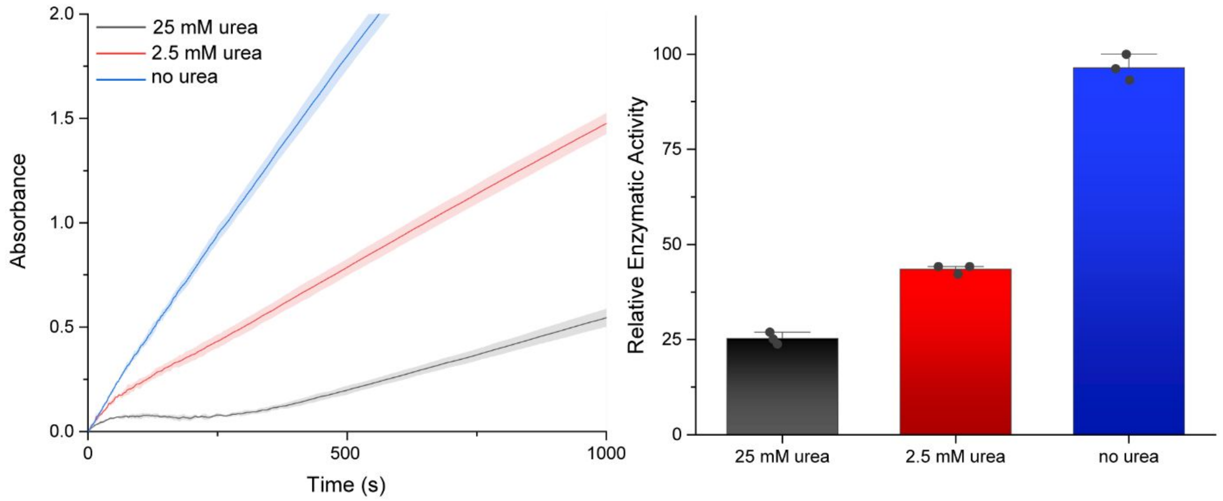

Figure S12. Influence of urea on the non-linear behavior of urease-HRP loaded BCNs in 5 mM phosphate buffer (left graph). The slopes were determined for the steady state activity (after the non-linear phase), and the relative activities were calculated (right graph).

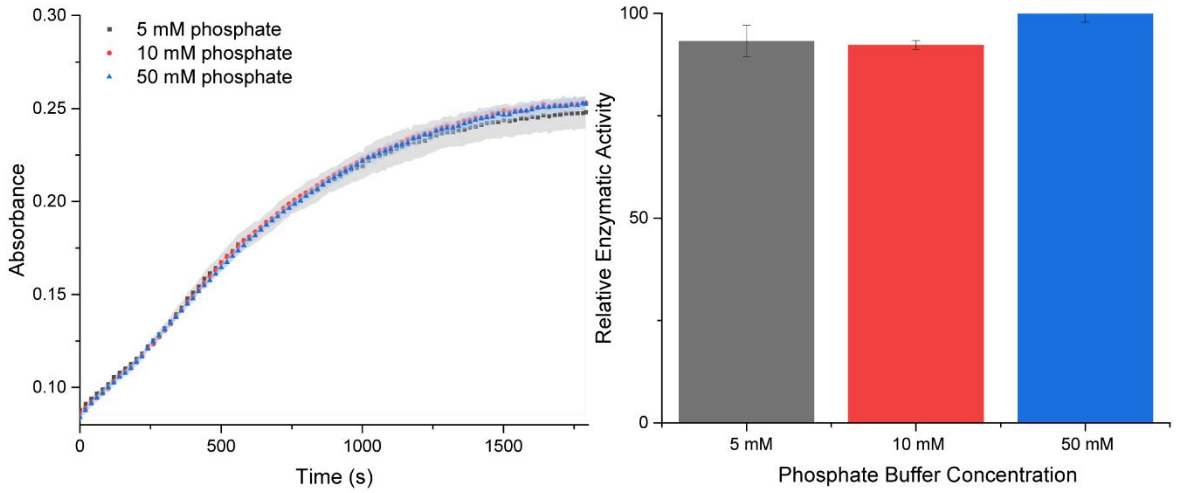

Figure S13. Influence of buffer capacity (phosphate buffer concentrations) on the HRP activity (left graph). The slopes were determined for the first 150 seconds in the linear regime, and the relative activities were calculated (right graph). Buffer capacity was varied from 5 to 50 mM phosphate. The relative activities show a negligible difference in relative enzymatic activity.

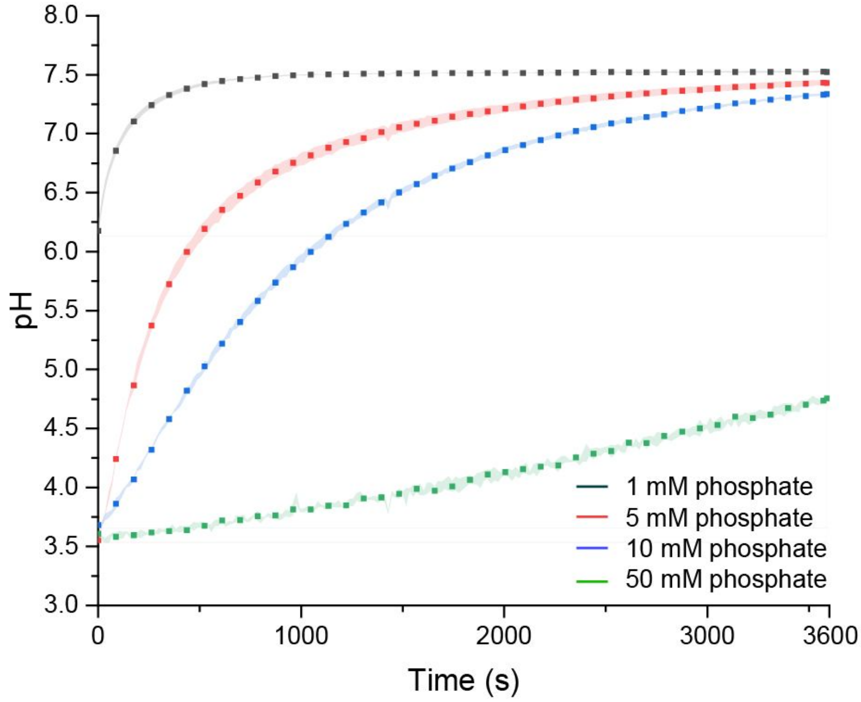

Figure S14. Bulk pH evolution of urease-HRP loaded BCNs using different buffer capacities. The urea concentration is 25 mM.

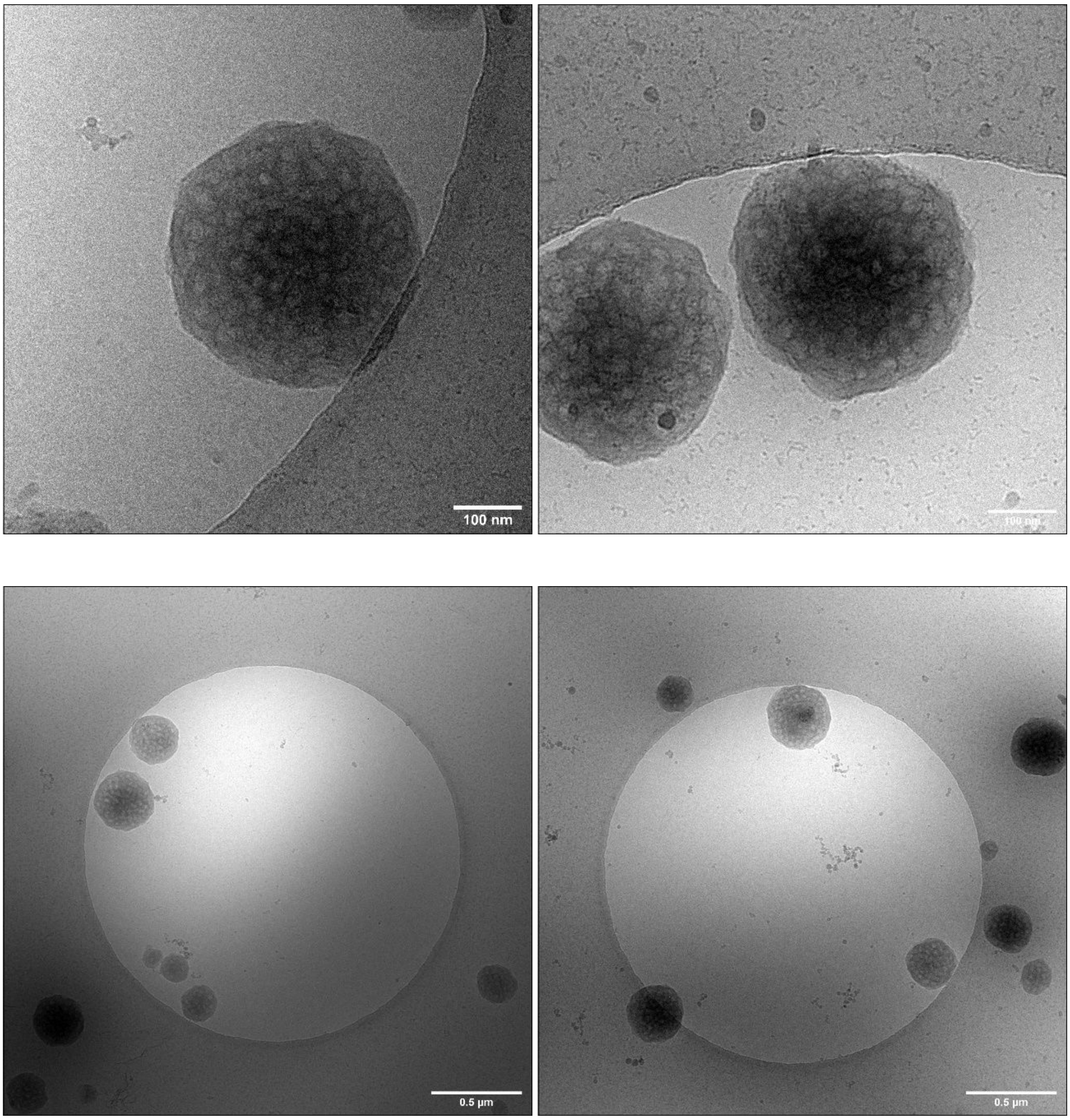

Figure S15. Additional Cryo-TEM images of bicontinuous nanospheres at pH 8. Scalebar represents 100 nm (top pictures) and 0.5 µm (bottom pictures).

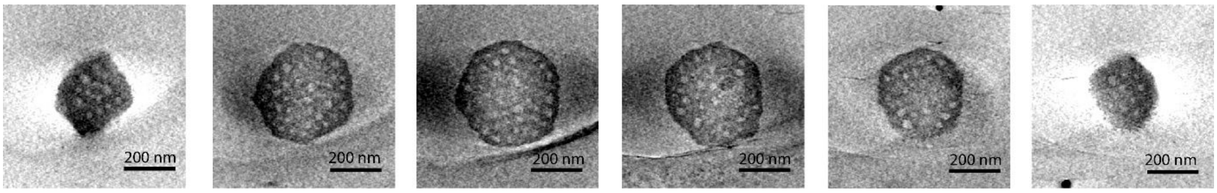

Figure S16. Additional Cryo-ET cross-sections of BCNs at pH 8. Scalebar represents 200 nm.

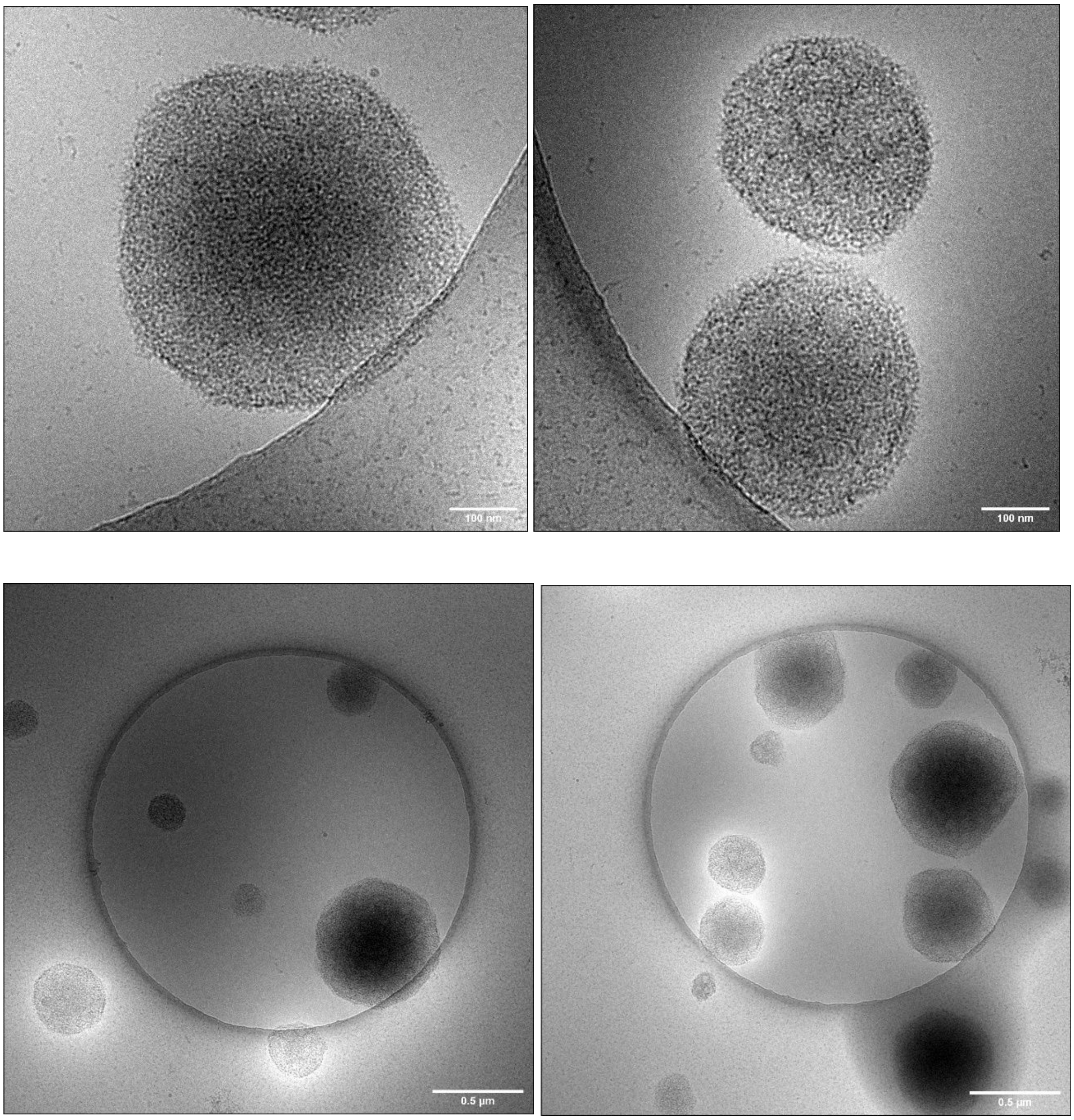

Figure S17. Additional Cryo-TEM images of bicontinuous nanospheres at pH 5. Scalebar represents 100 nm (top pictures) and 0.5 µm (bottom pictures).

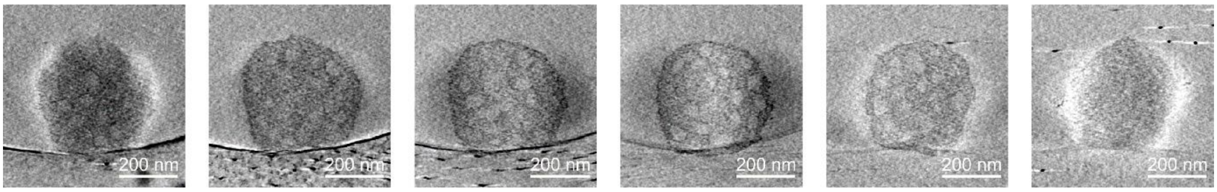

Figure S18. Additional Cryo-ET cross-sections of BCNs at pH 5. Scalebar represents 200 nm.

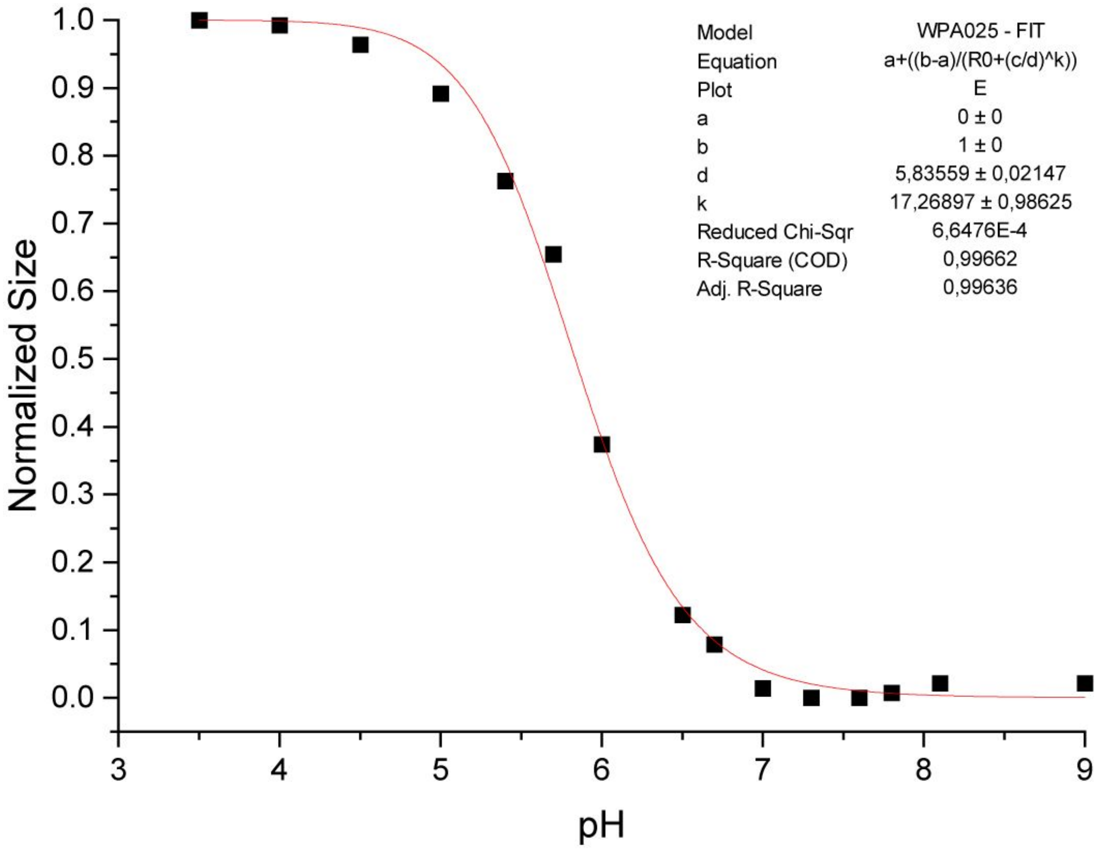

Figure S19. DLS pH-titration of the BCNs. The data is fitted according to the formula presented by Gumz et al. [1]

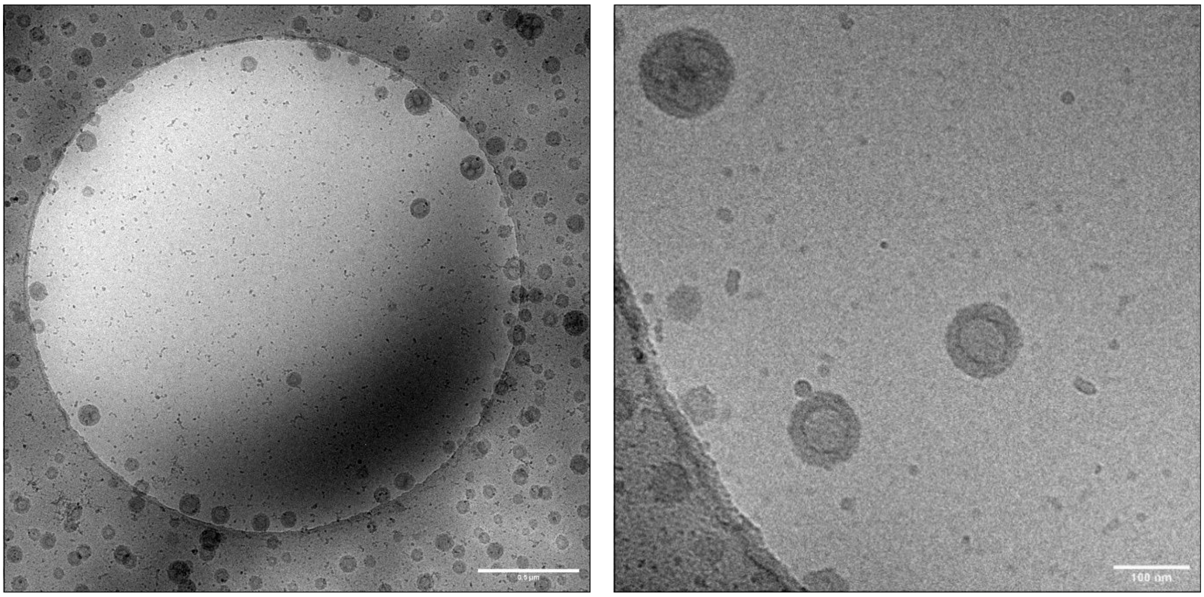

Figure S20. Cryo-TEM images of polymersomes at pH 8. Scalebar represents 500 nm and 100 nm respectively.

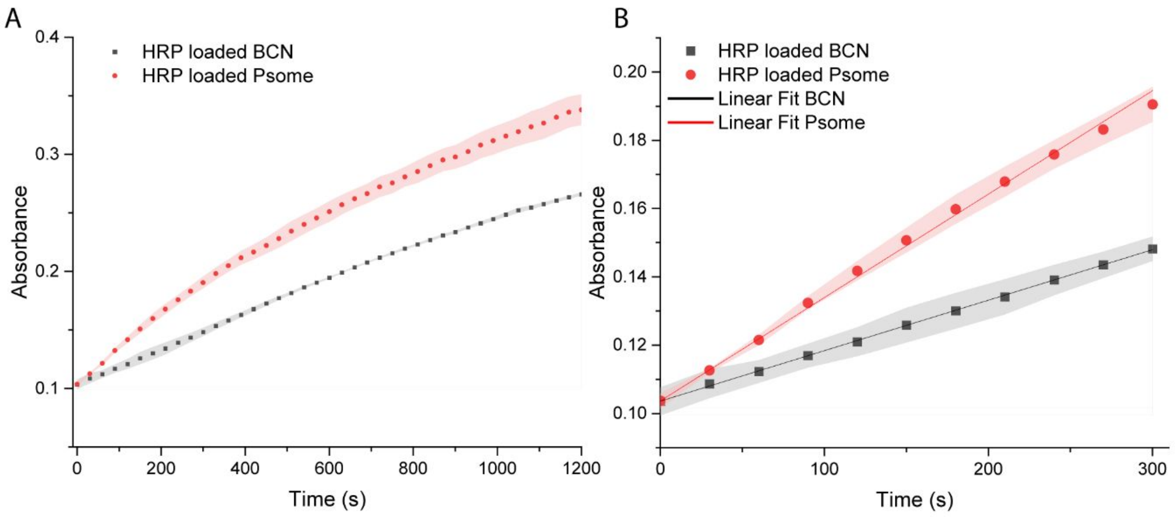

Figure S21. Probing the permeability of BCNs and polymersomes by following the conversion of ABTS in water. (A) Represents the absorbance time plots and (B) includes the linear fits from which the slopes were determined. The slope was determined in the linear regime. Please be noted that no urease or urea is added, and no pH effects occur.

From these slopes it was determined that polymersomes are approximately twice as permeable in comparison to the BCNs.

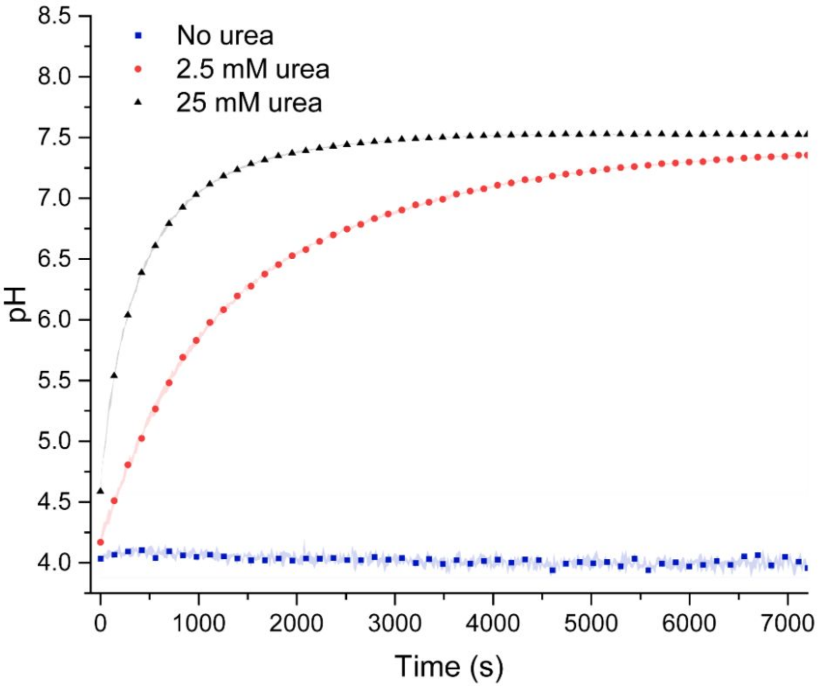

Figure S22. Bulk pH evolution curves of urease-HRP loaded polymersomes. (25 mM urea, 2 mM ABTS, 1 mM H_2O_2, 5 mM phosphate buffer)

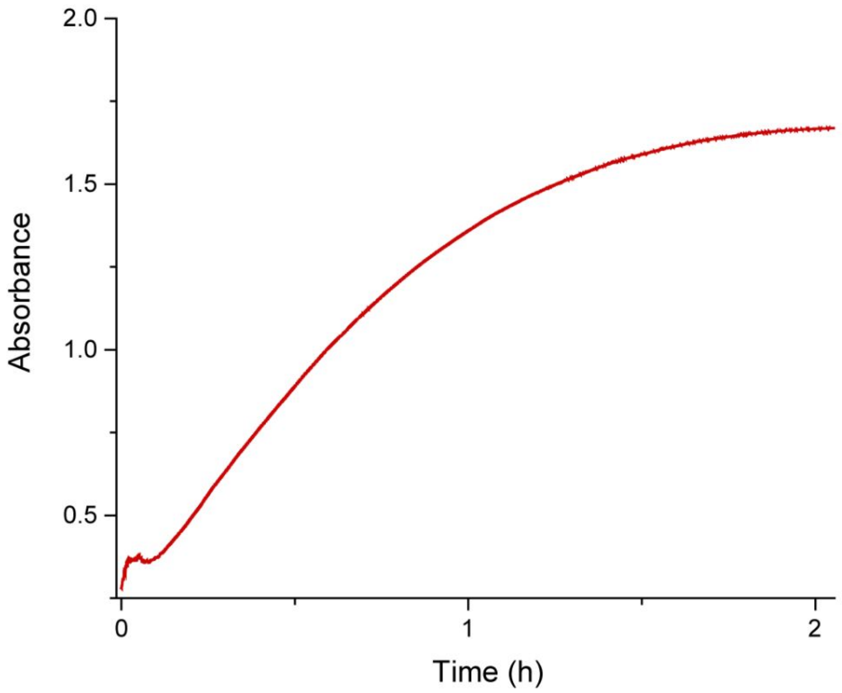

Figure S23. Complete cycle of urease-HRP loaded BCNs. The plateau at t=2h indicates the completion of the urease-feedback loop, rendering the BCNs impermeable (pH=pH*).

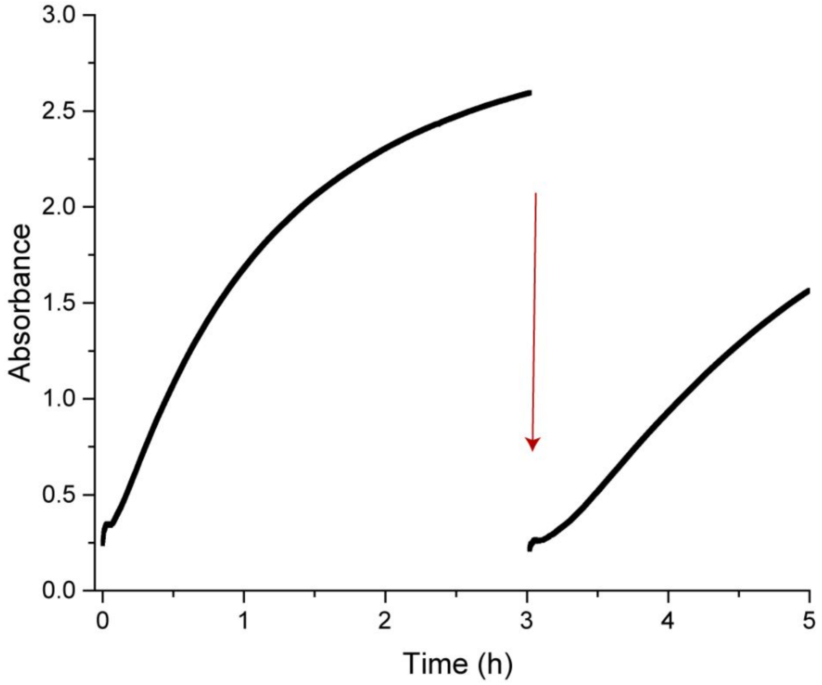

Figure S24. First and second cycle of urease-HRP loaded BCNs. After the first cycle, the BCNs are spin filtered using a 0.1 μm filter, and resuspended in 5 mM phosphate, pH 5 to equilibrate local & bulk pH.

Bibliography SI

[1] Gumz, H.; Lai, T. H.; Voit, B.; Appelhans, D. Fine-Tuning the PH Response of Polymersomes for Mimicking and Controlling the Cell Membrane Functionality. *Polym Chem* **2017**, *8* (19), 2904–2908. https://doi.org/10.1039/c7py00089h.
